# Heterocyclic π-linkers for reduced energy dissipation in symmetrical IDT-core-based non-fullerene acceptors: a route to efficient organic solar cells

**DOI:** 10.1039/d5na00680e

**Published:** 2025-10-16

**Authors:** Hina Naeem, Tahani A. Alrebdi, Karrar Hazim Salem, Muhammad Imran, Abdullah Almohammedi, Mohamed S. Soliman, Hira Naeem, Muhammad Faizan, Syed Muhammad Kazim Abbas Naqvi, Rasheed Ahmad Khera

**Affiliations:** a Department of Chemistry, University of Agriculture Faisalabad 38000 Pakistan rasheedahmadkhera@yahoo.com; b Department of Physics, College of Science, Princess Nourah bint Abdulrahman University P.O. Box 84428 Riyadh 11671 Saudi Arabia; c College of Medical and Health Technologies, Al-Zahraa University for Women Karbala Iraq; d Department of Chemistry, Faculty of Science, Research Center for Advanced Materials Science (RCAMS), King Khalid University P.O. Box 960 Abha 61421 Saudi Arabia; e Department of Physics, Faculty of Science, Islamic University of Madinah Madinah Saudi Arabia; f Department of Electrical Engineering, College of Engineering, Taif University Taif 21944 Saudi Arabia; g Department of Chemistry, GC-Women University Faisalabad Pakistan; h School of Materials Science and Engineering Jilin University Changchun China; i Faculty of Materials Science, Shenzhen MSU-BIT University Shenzhen 518115 China kazim72145@gmail.com; j Platform for Applied Nanophotonics, Institute of Advanced Interdisciplinary Technology, Shenzhen MSU-BIT University Shenzhen 518115 China

## Abstract

Achieving high power conversion efficiency (PCE) remains a major challenge in advancing organic solar cells (OSCs). In the field of organic photovoltaics (OPVs), substantial progress has been made in tuning molecular structures to enhance the PCE, yet innovative material design strategies targeting improved efficiency are still urgently needed. In this work, five novel A-π-D-π-A structured non-fullerene acceptor molecules (IDT1–IDT5) based on the IDT-ED-4F core are designed using density functional theory (DFT) and time-dependent DFT (TD-DFT) methods to explore their optoelectronic properties in both gas and solvent phases. Among these, IDT3 exhibited the lowest energy gap (*E*_g_ = 1.35 eV), the lowest electron reorganization energy (*λ*_e_ = 0.00578 eV), and a high absorption maximum, indicating its strong potential for efficient photon harvesting and charge transport. IDT1 showed the highest dipole moment in both gas (6.81 D) and solvent phases (7.59 D), which enhances its charge separation capability, while its high fill factor (FF = 90.93%) suggests improved carrier collection and device stability. The theoretical open circuit voltage (*V*_oc_) calculations revealed that IDT1 achieved the highest *V*_oc_ value of 1.40 V. Exciton binding energy (*E*_b_) analysis indicated that IDT3 had the lowest *E*_b_ value (0.14 eV), implying efficient exciton dissociation. Transition density matrix (TDM) and reduced density gradient (RDG) analyses confirmed effective intramolecular charge transfer (ICT) and stable non-covalent interactions within these molecules. Compared to the reference IDT-ED-4F molecule, all newly designed derivatives displayed reduced bandgap (*E*_g_), significant redshifted absorption, and enhanced charge mobilities. Overall, these results demonstrate that the newly developed IDT based molecules possess superior optoelectronic properties, establishing them as promising candidates for high efficiency next generation OSC applications.

## Abstract 2

Heterocyclic π-linker design in IDT-based acceptors enables efficient charge transfer, low bandgaps, and enhanced OSC performance.

## Graphical abstract

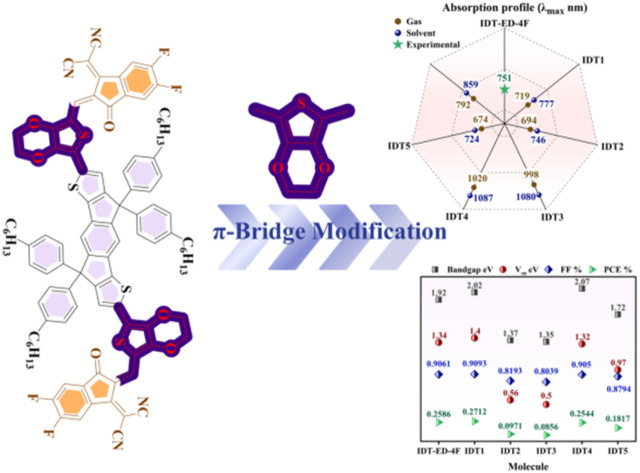

## Introduction

Driven by distinct benefits of organic solar cells (OSCs) over conventional silicon based solar cells, OSCs have emerged as a key component of modern solar energy technologies. These advantages include their lightweight and flexible structures, tunable bandgaps (*E*_g_), and ability to be manufactured through solution processing, making them ideal for applications such as flexible electronics, wearable devices, and semi-transparent solar panels.^1–4^ With the increase in global energy consumption and increasing environmental issues, OSCs have received much attention as a clean, flexible, and scalable alternative to conventional photovoltaics (PVs).^5,6^ Progress has been made in the efficiency, stability and application of solar cells since they were first developed in the early 19th century.^7^ In the past few years, organic semiconducting materials have drawn more and more attention for their good photophysical properties, structure variety, and multi-molecular tunability, enabling superior performance that overcomes the inherent limitations of inorganic solar cells.^8–10^ One key factor that has led to the swift advancement of OSC performance in recent years is the advent of non-fullerene acceptors (NFAs), which bring new opportunities in molecular design and device engineering.^11,12^ In contrast to conventional fullerene-based acceptors, NFAs possess better thermal and photochemical stability as well as broader and stronger absorption in the near-infrared region, and the rational design of electronic structures with energy levels matching those of donor polymers contributed to the high device performance.^13,14^ These properties have been combined to overcome key limitations of fullerenes and have resulted in record power conversion efficiencies for OSCs.

Among the strategies to improve NFA performance, molecular design of the photoactive layer is still an important aspect. Progress in computational chemistry has made it possible for the rational design and pre-synthetic optimization of NFAs to be achieved by predicting their electronic, optical, and structural properties.^15,16^ Structural modification strategies for NFAs include end-group engineering, side-chain optimization, central core modification, and π-spacer modification.^17,18^ In particular, π-spacer modification has gained attention due to its multifaceted benefits. It extends the conjugation length, enhances intramolecular charge transfer (ICT), increases molecular planarity and rigidity, and modulates frontier orbital energies for better donor–acceptor alignment.^19^ Furthermore, π-spacers improve intermolecular π–π stacking interactions and molecular packing in blend films, facilitating efficient charge transport while reducing recombination losses.^20,21^ As a result, A-π-D-π-A type molecular architectures incorporating π-spacers have demonstrated enhanced charge separation, balanced carrier mobility, and broad, intense absorption, leading to improved OSC performance.^22^ Carefully designed π-bridges not only broaden visible light absorption but also promote favourable morphological features that underpin high device efficiencies.^23^ A prominent example is the IDT-ED-4F molecule synthesized by S. J. Jeon *et al.*, incorporating 2,3-dihydrothieno[3,4-b][1,4]dioxine (EDOT) as a π-bridge. This molecular design led to an upshifted LUMO level and a highly planar backbone stabilized by non-covalent conformational locking, resulting in an impressive PCE of 10.4%.^24^ Its strong electron withdrawing end groups attached symmetrically to the IDT core enabled broad absorption (*λ*_max_ = 751 nm) and high charge mobility, establishing IDT core based NFAs as versatile acceptors compatible with various donor materials. Despite these efforts, the existing library for modification of the π-bridges within the IDT core in NFAs is quite limited, which hampers further tuning of their electronic structures, absorption properties, and charge transport properties. Moreover, systematic studies exploring the effects of different heterocyclic π-bridges on the OE behaviour and PV performance of IDT-core NFAs are still lacking. Addressing this knowledge gap is essential to develop NFAs with tailored electronic properties and superior device performance.

Recent DFT studies have shed valuable light on how molecular design strategies can be used to improve the performance of non-fullerene acceptors (NFAs). BTPT-OD derivatives have shown that careful tuning of the core and terminal groups can narrow the energy gap, shift absorption to longer wavelengths, and optimize donor–acceptor energy alignment, all of which support higher device efficiencies.^25^ Other investigations on fluorene-core NFAs have highlighted clear links between the π-bridge, end-group modifications and key parameters such as reorganization energy, absorption maxima, and open-circuit voltage. These studies emphasize that lowering the reorganization energy while strengthening ICT is an effective route to boosting both charge transport and photogeneration.^26^ Heavy atom substitution in CB16-type NFAs has been explored as a way to intensify charge transfer and tailor excited-state properties, providing another pathway to enhance photovoltaic performance.^27^ Motivated by these advances, the present work explores heterocyclic π-linkers symmetrically incorporated around an IDT core, with the goal of reducing energy losses, reinforcing charge transfer, and maintaining favourable planarity and packing. To achieve this, we designed five new derivatives of IDT-ED-4F with diverse π-bridges to systematically evaluate their optoelectronic and photovoltaic performances. These π-bridges consist of heterocyclic units such as 1,3,4-oxadiazole, thiazolo[5,4-*d*]thiazole, 1,3,5-triazine, 1,3,4-thiadiazole and methyl-substituted benzothiadiazole, each adding distinct electronic and geometrical features. 1,3,4-Oxadiazole and 1,3,4-thiadiazole have strong electron-withdrawing effects and show the planar conformation of the molecule, thiazolo[5,4-*d*]thiazole ensures the rigid π-conjugation and excellent charge mobility, triazine provides strong electron deficiency along with nitrogen rich aromaticity, and methyl group substituted benzothiadiazole supplies electron-withdrawing ability as well as enhanced molecular stability. The introduction of these π-bridges is expected to increase the conjugation, strengthen ICT, reduce the reorganization energy, broaden the absorption, improve the molecular packing and facilitate charge transport, which are favourable for achieving high performance OSCs. In this study, newly designed molecules have been examined using density functional theory (DFT)^28^ and time-dependent DFT (TD-DFT)^29^ calculations to evaluate their electronic structures, optical properties, electron–hole recombination efficiencies (RE), and PV performance. This study provides comprehensive insights into the rational design of π-bridge engineered NFAs, advancing the progress of next generation high performance OSCs and offering new molecular design strategies for future OPV devices.

## Theoretical methods

All the calculations were carried out with the Gaussian 09 simulation package.^30^ The molecular structures and orbitals were analysed using GaussView 6.0.^31^ The reference molecule (IDT-ED-4F) was first optimized with four different functionals, namely B3LYP,^32^ CAM-B3LYP,^33^ MPW1PW91,^34^ and WB97XD.^35^ Among these, the B3LYP functional yielded the best correlation with the experimentally reported absorption maximum of the IDT-ED-4F (751),^24^ producing a theoretical value of 777 nm. Based on this result, designed molecules were subsequently optimized using the B3LYP/6-31G(d,p) functional basis set.^36^ To analyse the electronic structure and density of states (DOS), PyMOlyze-1.1 (ref. 37) was employed for plotting and visualization. Additionally, key molecular properties including dipole moment (*D*),^38^ binding energies (*E*_b_),^39^ and open circuit voltage (*V*_oc_)^40^ were computed to assess the charge distribution, interfacial compatibility, and PV potential of the designed molecules. The transition density matrix (TDM)^41^ was calculated using Multiwfn software^42^ to clarify the character and magnitude of charge transfer from donor to acceptor fragments. Also, the reorganization energy (*λ*), which is an important parameter that determines the charge mobility, was estimated for both electrons and holes.^43,44^ This parameter is directly correlated with the efficiency of charge transport and is calculated according to Marcus theory.^45^ The reorganization energies were obtained through the following expression:1
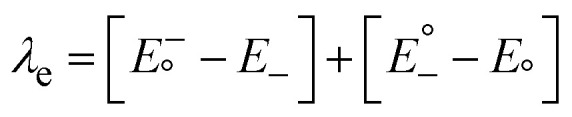
2
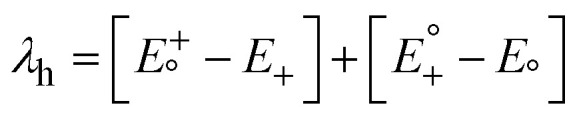
where *λ*_e_ is the reorganization energy of an electron, associated with the structural relaxation during electron transfer, while *λ*_h_ is the reorganization energy of a hole, associated with the structural relaxation during hole transfer. *E*_−_ is the energy of the relaxed anion and *E*^°^_−_ is the single point energy of the anion using the optimized geometry of the neutral species. Likewise, *E*_+_ is the total energy of the optimized cationic species and *E*^°^_+_ is the single point energy of the cation in the neutral geometry. These energy values were subsequently utilized in the Marcus theory equations to calculate the reorganization electrons (*λ*_e_) and holes (*λ*_h_) that correlate with charge transport across the organic semiconducting materials.

## Optoelectronic (OE) characteristics

### Structural optimization

**Fig. 1:**
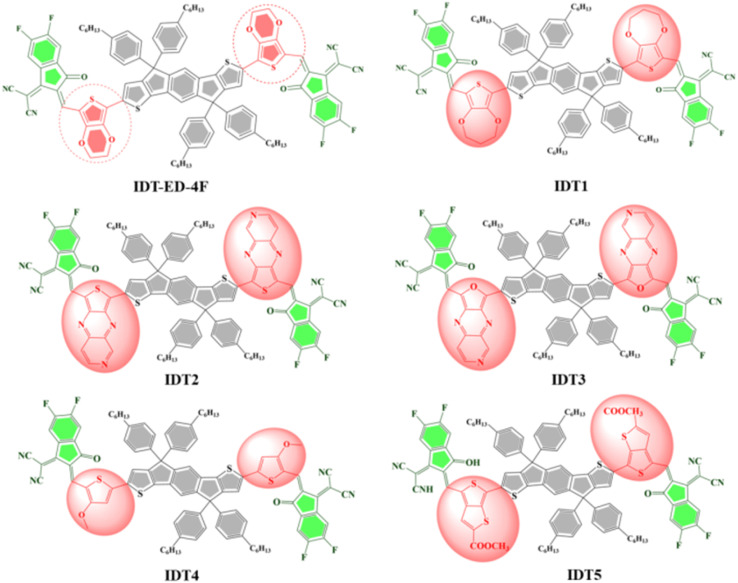
Representation of geometry of R and designed molecules (IDT1–IDT5).

The geometry of the IDT-ED-4F was optimized in the solvent phase by DFT approaches, such as B3LYP, CAM-B3LYP, WB97XD and MPW1PW91, in order to test the performance of different DFT functionals. Of these, B3LYP was found to be the most suitable functional as it provided calculated absorption maxima of 777 nm and 719 nm in the gas phase, which are relatively close to the experimental maximum of 751 nm, as shown in Fig. S1. Based on this validation, the B3LYP functional was employed for the optimization of all molecules in the study. IDT-ED-4F was structurally modified by introducing different heterocyclic π-bridges between the donor core and terminal acceptor groups. These modifications resulted in five novel derivatives (IDT1–IDT5), each incorporating a distinct π-bridge while preserving the central IDT core and terminal electron-withdrawing groups. Consequently, all designed molecules maintain an acceptor-π-donor-π-acceptor (A-π-D-π-A) configuration, as illustrated in Fig. 1. All designed molecules were then optimized using density functional theory (DFT) at the B3LYP/6-31G(d,p) level to investigate the influence of π-bridge insertion on their electronic structures, optical properties, and potential application in organic PV devices.

The photoelectric characteristics of the designed IDT derivatives are significantly influenced by their bond lengths and dihedral angles.^46^ The dihedral angles between the donor, π-bridge, and acceptor units explain the spatial arrangement and torsional behaviour of these molecules.^47^ Bond angles and bond lengths between the π-bridge, donor, and acceptor subunits were analysed from the optimized geometries to gain insights into their structural organization and electronic interactions. The corresponding values are summarized in Table 1. A single C–C bond generally exhibits a bond length of approximately 1.54 Å, while a C=C double bond is typically around 1.34 Å. The distances between the donor, π-bridge, and acceptor units between two adjacent atoms (*L*_1_ and *L*_2_) in the designed molecules are in the range of 1.39–1.45 Å, suggesting strong delocalized π-conjugation across the molecular framework. This intermediate bond distance implies strong electronic coupling and conjugation, which are important for facilitating charge transport in these structures. Moreover, the dihedral (torsional) angles in between the donor, π-bridge, and acceptor units range in between 0.00° and 4.56°, with IDT3 displaying the minimum torsional angle (0.00°), which suggests a more planar configuration. Whereas the highest torsional angle (4.56°) is found in IDT4, and hence it might be suggested that symmetry is lost and delocalization in such a coplanar arrangement is preferentially adjusted. Small torsional angles in this case are typically associated with planar systems that promote efficient π-electron delocalization and hence high degrees of conjugation. These structural parameters are essential for efficient charge transfer in organic PV cells. The torsion angles and inter-atomic distances are presented in Fig. S2.

**Table 1:** Structural parameters of the IDT-ED-4F and designed molecules derived from optimized geometries

Molecules	Bond length (*L*_c–c_) (Å)		Bond angle (*θ*^°^)		MPP (Å)	SDP (Å)
	*L*_1_	*L*_2_	*θ*_1_^°^	*θ*_2_^°^		
IDT-ED-4F	1.40	1.43	0.67	0.65	1.624	9.103
IDT1	1.40	1.41	0.07	0.22	1.489	9.113
IDT2	1.39	1.40	0.02	0.21	1.488	9.113
IDT3	1.40	1.43	0.03	0.00	1.625	9.104
IDT4	1.45	1.43	4.56	0.20	1.812	10.252
IDT5	1.41	1.43	0.06	0.05	1.545	9.097

Molecular planarity significantly influences the OE performance of organic semiconductors. In this study, the molecular planarity parameter (MPP) and span of deviation from the plane (SDP) were employed to quantitatively access the geometric planarity of DT-ED-4F and designed molecules (IDT1–IDT5). MPP and SDP values were calculated using Multiwfn 3.8, and molecular visualizations were prepared using VMD 1.9.3.^48^ A lower MPP value indicates a flatter configuration, which promotes effective π–π stacking and facilitates charge mobility in OSCs.^49^ SDP quantifies the maximum vertical range of atomic displacements relative to the fitted plane, describing the extent of local distortion. Thus, MPP and SDP in combination provide a comprehensive assessment of molecular coplanarity, a key determinant of intermolecular interactions, charge delocalization, and overall device performance.^50^ The calculated MPP values for IDT-ED-4F and the designed molecules are 1.624, 1.489, 1.488, 1.625, 1.812, and 1.545 Å, respectively. IDT2 exhibits the lowest MPP value (1.488 Å), indicating superior structural planarity, while IDT4 shows the highest MPP value (1.812 Å), suggesting greater torsion within its molecular framework. The corresponding SDP values are 9.103, 9.113, 9.113, 9.104, 10.252, and 9.097 Å, confirming that IDT4 exhibits slightly higher deviation from planarity compared to other derivatives. These geometrical parameters are tabulated in Table 1 and illustrated in Fig. S3. In the visualizations, shades of red and blue signify segments below and above the molecular plane, respectively. For example, the 1,4-dioxepane rings in the π-spacer of IDT1 are displayed in blue, indicating their position above the fitted plane. Overall, all designed molecules demonstrate near-planar structures, which are favourable for optimal charge carrier mobility and high-performance organic solar cell applications.

### Optical absorption characteristics

The optical characteristics of molecules are crucial in determining their PV performance in OSCs. The excited state parameters have gained wide acceptance in studying the mechanisms underlying photocurrent generation and their relationship to spectral features. The absorption spectra of the IDT-ED-4F and the designed molecules were calculated using TD-DFT at the B3LYP/6-31G(d,p) level of theory. The calculated oscillator strength (*f*), dipole moment (*D*), and maximum absorption wavelength (*λ*_max_) in the gas phase and solvent phase (chloroform) are tabulated in Tables S1 and S2, respectively. The π-bridges introduced between the donor and acceptor parts of these molecules possess strong electron-withdrawing abilities, which caused their absorption peaks to redshift towards longer wavelengths and narrowed their band gaps. This redshift is attributed to the elevated electron-attracting effect of the π-bridges, which influences the HOMO and LUMO energy levels and facilitates the ICT transitions. There is an inverse relationship between *λ*_max_ and *E*_g_. Molecules with lower *E*_g_ values exhibit absorption spectra shifted towards longer wavelengths (redshift), as also observed in this study.^51^

**Fig. 2:**
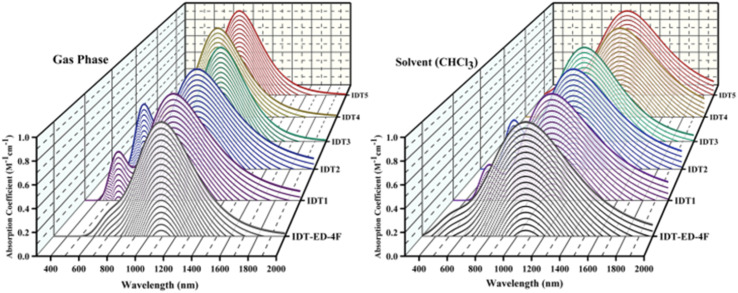
Absorption spectra for IDT-ED-4F and designed molecules (IDT1–IDT5).

The calculated *λ*_max_ values of the designed molecules range between 674 and 1087 nm in the solvent phase and lie close to those of IDT-ED-4F. All of the designed derivatives have redshifted absorption spectra compared to IDT-ED-4F. The highest redshift was found for IDT3 with a *λ*_max_ of 1087 nm, which was attributable to the maximum wavelength of IDT3 being most strongly affected by the substitution of its unique π-bridge. The *λ*_max_ values in the solvent phase followed the order: IDT3 (1087 nm) > IDT2 (1080 nm) > IDT5 (859 nm) > IDT1 (746 nm) > IDT4 (724 nm). In the gas phase, the order was IDT3 (1020 nm) > IDT2 (998 nm) > IDT5 (792 nm) > IDT1 (694 nm) > IDT4 (674 nm). All the designed molecules had wider absorption spectra than IDT-ED-4F in both phases. These broadened and redshifted spectra suggest a stronger photon harvesting potential for OSC applications. The results demonstrate that all designed molecules possess enhanced absorption characteristics compared to the reference molecule. Their strong and broad absorption in the visible to near infrared region suggests that these newly designed molecules are promising candidates as active materials for high performance OSCs. The absorption spectra of IDT1–IDT5 and designed molecules are illustrated in Fig. 2.

### The frontier molecular orbitals (FMOs)

Molecular orbitals are fundamental descriptors in quantum chemistry used to examine the charge transfer, molecular interactions, and electronic distribution, particularly in the design of organic OE materials. In the context of OSCs, FMOs are directly related to light absorption, photoexcitation, and the efficiency of charge transport. The alignment of HOMO and LUMO levels plays a key role in photovoltaic performance. In donor–acceptor systems, the LUMO energy of the acceptor must be suitably below the donor HOMO to enable efficient exciton dissociation and electron transfer. A deeper LUMO generally enhances electron affinity and electron transport, while an excessively low LUMO can increase energy losses.^52^ Similarly, the energy offset between the donor HOMO and acceptor LUMO largely determines the achievable *V*_oc_ with a larger offset supporting higher photovoltage generation.^53^ A smaller *E*_g_, which corresponds to higher softness and lower hardness, facilitates electronic transitions and supports efficient photon absorption and electron acceptance.^54^ Upon photoexcitation, electrons shift from the HOMO to the LUMO, transitioning from the ground state (*S*_0_) to the excited state (*S*_1_). Typically, a smaller *E*_g_ enhances ICT.^55^ Moreover, the presence of greater electron density in the HOMO on the π-bridge and LUMO localization on the acceptor units promotes directional charge transfer toward the terminal acceptors upon excitation. In this study, the HOMO–LUMO energy levels and corresponding *E*_g_ values for the IDT-ED-4F and the designed derivatives were calculated to assess their electronic structures. These values are summarized in Table S3. The results demonstrated that designed molecules in which strong electron withdrawing π-bridges were incorporated in the molecular structure had a lower LUMO energy level resulting in a narrower *E*_g_ compared to IDT-ED-4F. A smaller *E*_g_ signifies better electronic coupling between the donor and acceptor, which is beneficial for the efficient charge transfer and the OE performance of the molecules. The calculated *E*_g_ values in ascending order are: IDT3 (1.35 eV) < IDT2 (1.37 eV) < IDT5 (1.72 eV) < IDT1 (2.02 eV) < IDT4 (2.07 eV) < IDT-ED-4F (1.92 eV).

**Fig. 3:**
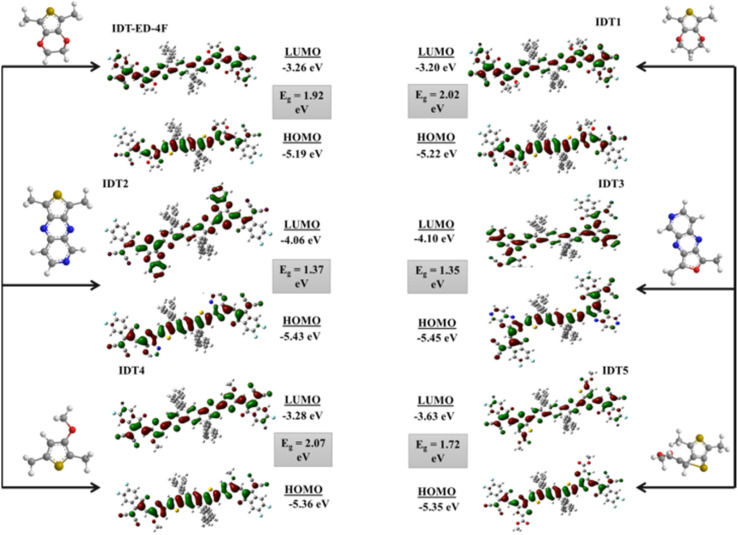
FMO diagram of IDT-ED-4F and designed molecules (IDT1–IDT5).

Among the designed molecules, IDT3 has the lowest *E*_g_ value owing to the introduction of a strong electron withdrawing π-bridge that significantly decreases the LUMO energy level and enhances ICT. IDT2 also presents an exceptionally small *E*_g_ (1.37 eV) due to its strong π-bridge effect, which is beneficial for electron delocalization and thus narrowing of the excitation *E*_g_. The HOMO and LUMO energy levels of the IDT-ED-4F and designed molecules are presented in Table S3. The HOMO values vary from −5.19 to −5.45 eV and the LUMO values vary from −3.20 to −4.10 eV. The FMO distributions of the IDT-ED-4F and designed molecules are depicted in Fig. 3, illustrating that in the ground state, the HOMO is primarily delocalized over the donor core and π-bridge, while in the excited state, the LUMO becomes more localized on the terminal acceptor units. This spatial separation of HOMO and LUMO confirms efficient ICT upon excitation, which is a key feature for high performance OSCs. IDT-ED-4F exhibited a higher *E*_g_ compared to IDT2, IDT3, and IDT5, which can be attributed to the absence of strongly electron withdrawing π-bridges. Consequently, it faces more challenges in promoting the excitation of electrons from the HOMO to the LUMO. The reduced *E*_g_ values in the designed molecules indicate their enhanced potential as efficient active materials in OSCs due to improved photon absorption, stronger ICT, and superior charge transport characteristics.

### Density of states (DOS) analysis

**Fig. 4:**
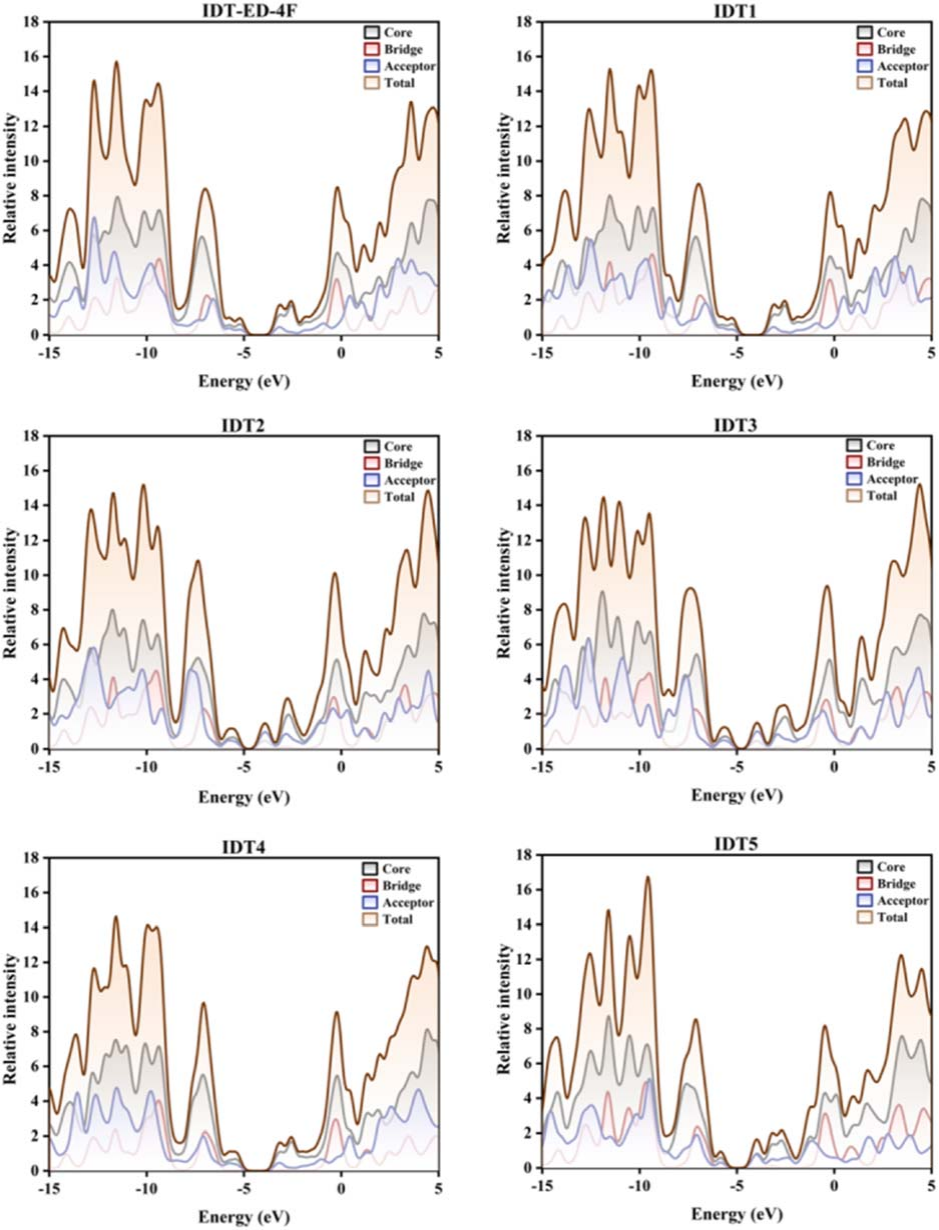
DOS spectra of the IDT-ED-4F and designed molecules, showing the contributions of the donor core, π-bridge, and terminal acceptor to the HOMO and LUMO regions.

The DOS analysis provides valuable insights into the distribution of energy levels within FMOs, elucidating the electronic transitions and absorption features associated with different molecular fragments, namely the donor core, π-bridge, and terminal acceptor units. To determine the specific contributions of each fragment to the overall electronic structure, the molecules were partitioned into three subunits: donor core, π-bridge, and terminal acceptor. The computed DOS profiles depicted in Fig. 4 reveal the extent to which each fragment contributes to the HOMO and LUMO energy levels.

Fig. 4 shows the DOS for the designed molecules IDT1, IDT2, IDT3, IDT4, and IDT5, along with the reference molecule IDT-ED-4F. These plots illustrate how the electronic states are distributed among the core, bridge, and acceptor fragments within an energy range from −15 eV to +5 eV, providing insight into their electronic structures and functional fragment contributions. For all designed molecules, the core fragment shows a dominant contribution, particularly in the deep valence region below −5 eV. This implicates that the core unit possesses excellent electron stability, acting as the backbone to support high efficient conjugation. The designed derivatives, in particular IDT2 and IDT3, display distinct and strong core contributions indicating increased structural rigidity and electronic stabilization when compared to the reference molecule IDT-ED-4F. The contribution of the π-bridge fragment is moderate throughout the valence band and has two significant peaks at the vicinity of HOMO levels (−5 eV to 0 eV). This suggests the essential role of the π-bridge for connecting the core unit and acceptor and for facilitating ICT. Importantly, not only the π-bridge contributions at the HOMO are stronger for the designed molecules but also, for instance, near the HOMO, in particular for the IDT2 and IDT3 molecules. This indicates improved π-electron delocalization and improved charge transport that are critical for efficient charge transport in OE devices. The acceptor fragment dominates close to the LUMO (0 to +5 eV), which is consistent with its electron-withdrawing nature. The designed molecules reveal strong acceptor contributions near the LUMO. This suggests that the designed molecules are successfully designed for improving electron-accepting properties.

In addition, the acceptor states are more widely distributed in the designed molecules, indicating a more extensive delocalization of electrons, enabling more efficient charge separation and transfer. The DOS calculations reveal that the designed molecules not only retain the electronic stability of the core units they bear but also show superior π-bridge contributions and higher acceptor contributions compared with the reference molecule. Higher π-bridge contributions in the designed molecules imply better ICT pathways, and larger values of acceptor contributions show higher electron affinity, which is important for efficient exciton dissociation and charge mobility in PV applications. DOS and partial DOS (PDOS) analyses indicate that the designed molecules have better electronic structures than the IDT-ED-4F. Optimized stability, promoted π-bridge mediated charge delocalization and significant acceptor contributions indicate that these designed molecules are more suitable for high performance OE devices with potential to achieve high charge mobility efficiency and device performance.

### Molecular electrostatic potential (MEP)

**Fig. 5:**
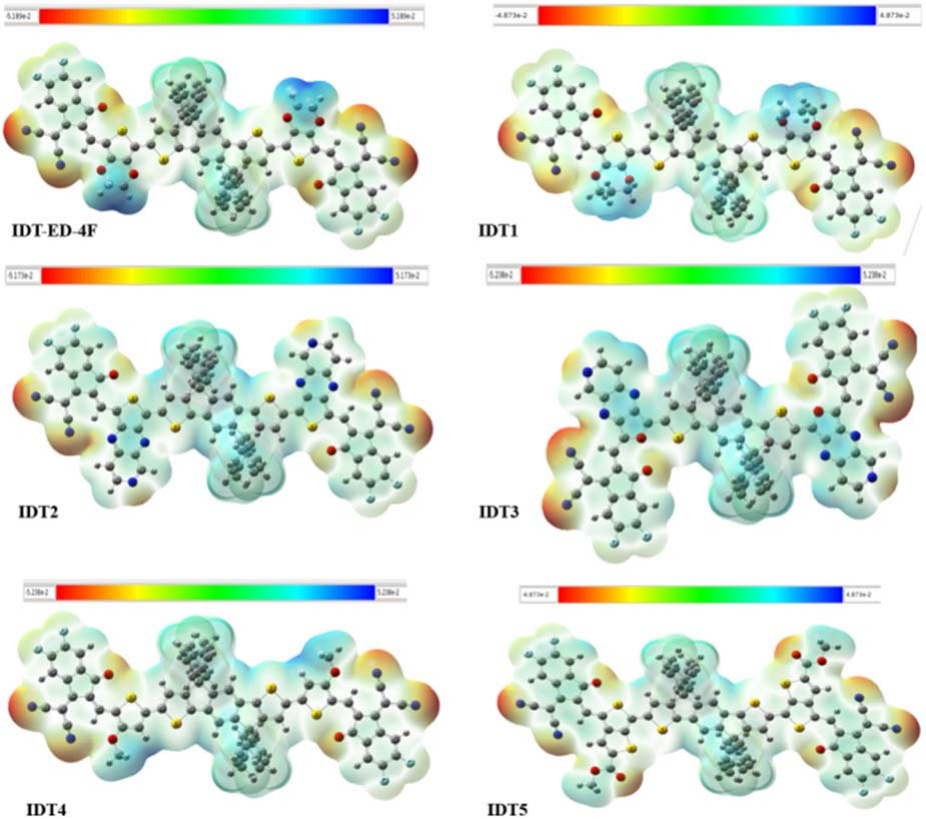
MEP surfaces of the IDT-ED-4F and designed molecules, highlighting regions of electron density.

MEP analysis is a valuable tool to visualize the electron distribution throughout a molecule, to locate electrophilic and nucleophilic sites. MEP maps provide an understanding of charge separation and intramolecular interactions, which are essential for the explanation of OE properties of organic semiconductors. In terms of electronic properties of OSCs, MEP analysis helps to understand about the directionality and the efficiency of pathways for charge transfer in the donor–acceptor system. The electrostatic potential on the molecular surface is colour coded to reflect the local charge environment. The red and yellow areas are generally predictable as the deformation density regions with negative electrostatic potential and electron rich (acceptor) sites, while the blue regions indicate positive electrostatic potential and electron deficient (donor) regions. Neutral potential regions are indicated in green. The electrostatic potential generally follows the gradient: red < yellow < green < cyan < blue, moving from the most negative to the most positive potential.^56^Fig. 5 displays the MEP surfaces of the IDT-ED-4F and the designed derivatives. In all molecules, the central donor cores are predominantly marked by blue regions, indicating electron-deficient zones, while the terminal acceptor units feature red, yellow, and green areas, corresponding to electron rich sites. This pattern indicates a directional charge separation from the electron-donating core to the electron withdrawing ends, which supports efficient ICT upon excitation.

Among all designed molecules, IDT3 displays the most intense red zones at the terminal ends. This is attributed to the strong electron-withdrawing nature of the oxygen and nitrogen atoms present in its acceptor groups, which pull electron density towards the acceptor, enhancing charge separation and improving charge mobility within the molecule. This observation further supports the role of molecular structural design in modulating electronic properties for enhanced device performance. Overall, MEP analysis confirms that all designed molecules exhibit effective charge separation and favourable electron density distribution, which are essential characteristics for high performance photoactive materials in OSC applications.

### Transition density matrix (TDM) analysis

**Fig. 6:**
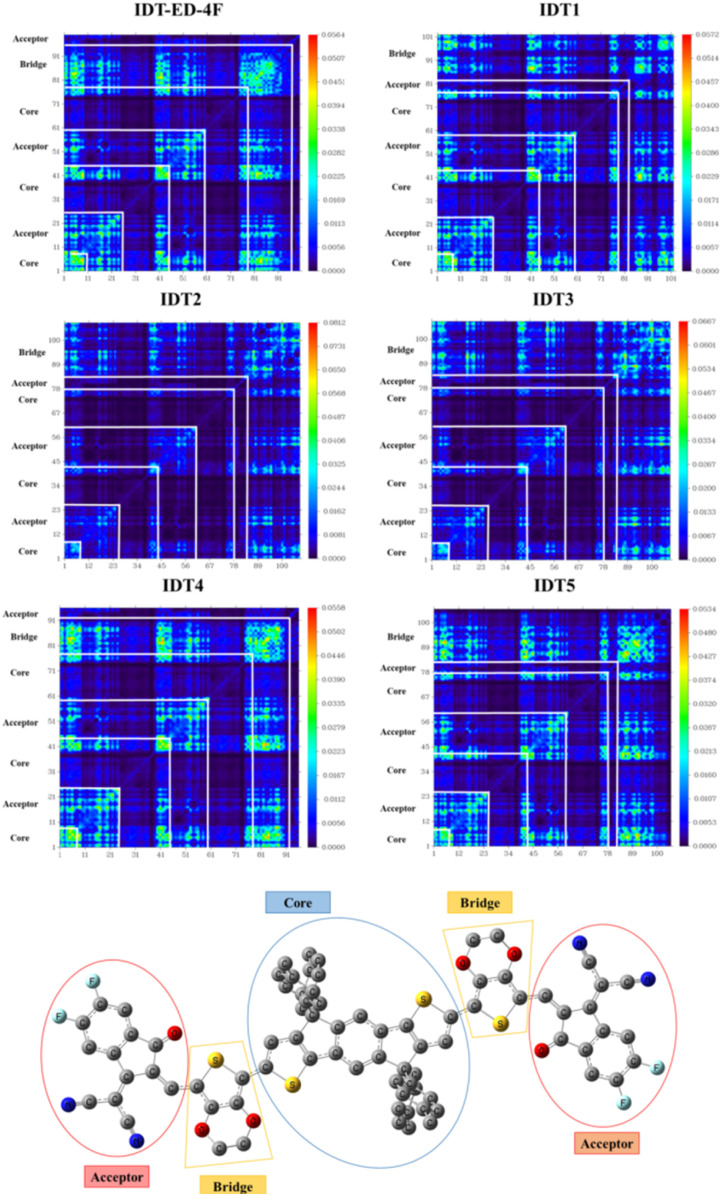
TDM map of IDT series showing the electron density distribution in the molecule.

TDM analysis is an effective approach to explore the excited state dynamics of the molecules and offer a detailed understanding on redistribution of electronic charge upon photoexcitation. It offers information on the spatial separation between electron and hole populations and is important in assessing the ICT efficiency and extent of electronic delocalization. In this study, TDM analysis was performed for the IDT-ED-4F and the designed derivatives to explore the direction and magnitude of charge movement following excitation. Hydrogen atoms were omitted from the analysis because of their negligible contribution to electronic transitions. The TDM plots presented in Fig. 6 display excitation characteristics using a colour mapped matrix. In these plots, the colour intensity represents the degree of electron–hole correlation. Red zones indicate high electron density accumulation, blue zones show minimal activity, and intermediate colours represent transitional regions. Diagonal elements correspond to localized excitations within the same region, while bright regions off the diagonal indicate charge transfer between distant molecular segments, suggesting effective long range ICT. All designed molecules exhibit charge migration from the donor core to the terminal acceptor units, indicating efficient charge delocalization. In particular, IDT3 shows distinct off-diagonal hotspots, suggesting stronger ICT behaviour compared to the IDT-ED-4F. These patterns demonstrate favourable electronic communication between the donor, π-bridge, and acceptor units, supporting the design strategy for improved charge separation. The TDM results shown in Fig. 6 confirm that the newly designed molecules have efficient ICT, which is an essential characteristic for their application as high performance photoactive materials in OSCs.

### Reorganization energy (*λ*)

Reorganization energy (*λ*) is a key parameter in the design and evaluation of high performance materials for OSCs. It is directly associated with the rate of charge transfer and, consequently, has a significant impact on the overall efficiency of OSC devices. Generally, there is an inverse relationship between *λ* and charge mobility, as a lower *λ* facilitates faster charge transport which is essential for achieving optimal PV performance. Reorganization energy comprises two components: internal reorganization energy (*λ*_int_) and external reorganization energy (*λ*_ext_). The internal component includes *λ*_h_ (associated with hole transport) and *λ*_e_ (associated with electron transport), while the external component accounts for the influence of the surrounding environment. In this study, only *λ*_int_ is considered, given the focus on isolated molecular systems. The *λ*_int_ reflects the structural reconfiguration that occurs within a molecule as it transitions between neutral and charged states. According to Marcus theory, the rate of charge transfer increases as reorganization energy decreases, and it reaches a maximum when the driving force equals the *λ*. This theoretical framework highlights the direct connection between the molecular structure and charge transport efficiency in organic semiconductors. Specifically, *λ* reflects the structural adjustments required upon oxidation or reduction; *λ*_e_ corresponds to the geometry of the anionic state, while *λ*_h_ relates to the geometry of the cationic state. The *λ*_e_ and *λ*_h_ values for the IDT-ED-4F and the designed derivatives are listed in Table S4. It was found that all the designed molecules have higher *λ*_e_ values than the reference molecule IDT-ED-4F.

### Reduced density gradient analysis for noncovalent interactions

**Fig. 7:**
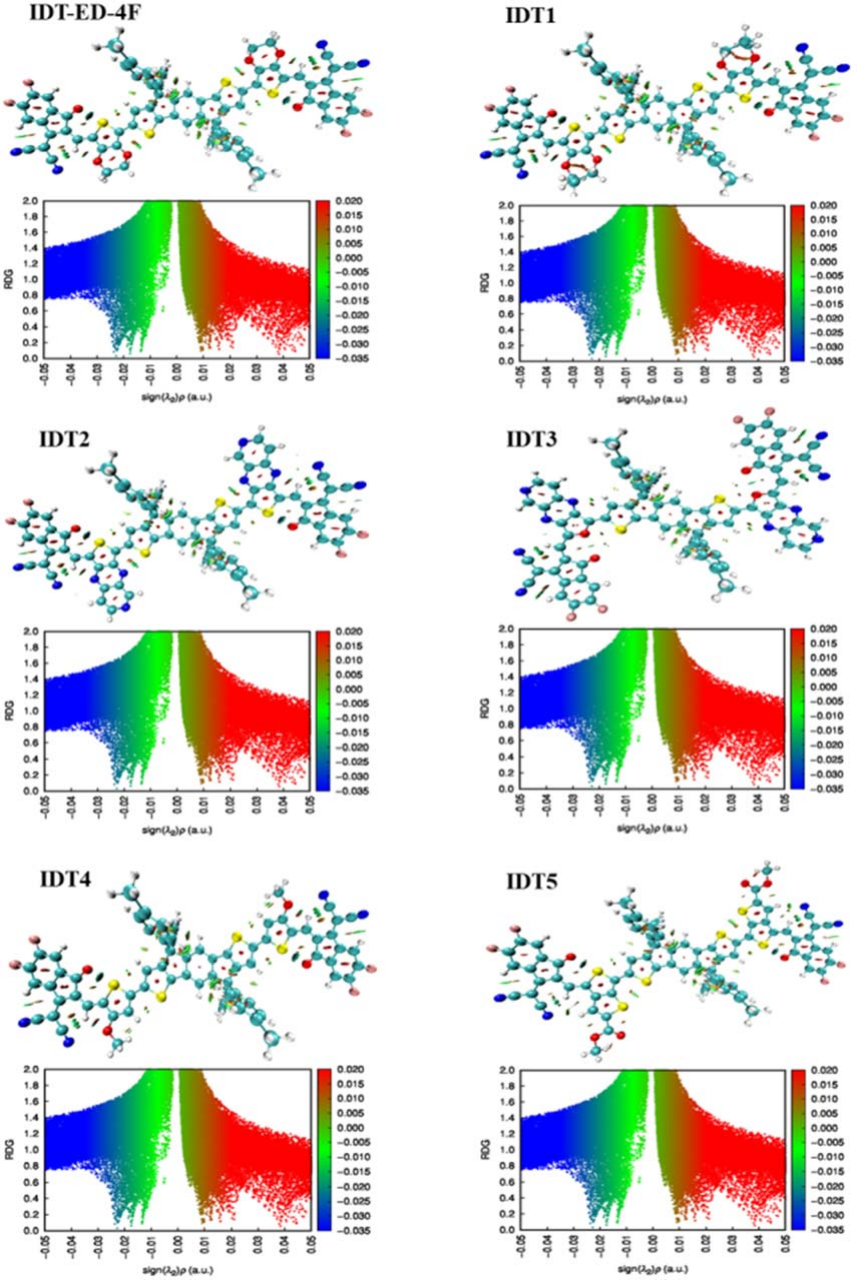
RDG plots showing NCIs in IDT-ED-4F and designed molecules.

Noncovalent interactions (NCIs) play an important role in determining the structural stability, conformation, and OE performance of OSC materials. While covalent bonds define the primary structure of molecules, NCIs such as hydrogen bonds, π–π stacking, van der Waals (vdW) interactions, and steric repulsion regulate molecular aggregation and tertiary structures.^57^ These interactions are influenced by the environment created by coordinated atoms and are crucial for maintaining the structure and function of organic semiconductors.^58^ Reduced density gradient (RDG) analysis is an important computational method used to investigate NCIs within molecules.^59^ It involves plotting the reduced density gradient as a function of electron density to identify weak interactions such as hydrogen bonding, vdW forces, and steric effects.^60^ The RDG function is calculated based on electron density and its gradient, where low electron density (*ρ*) and low reduced gradient (*s*) values indicate the presence of NCIs. Interactions can be classified as attractive or repulsive based on the sign of the second eigenvalue of the Hessian matrix (*λ*_2_). Attractive interactions (such as hydrogen bonding) are indicated by *λ*_2_ <0 and appear as blue regions, vdW interactions are shown in green, and repulsive steric interactions are indicated by *λ*_2_ >0 and appear in red. The visualization uses colour mapping to differentiate between these interactions. By default, RDG isosurfaces are plotted at a value of 0.5, and a high quality grid (0.016 Bohr) is used to ensure satisfactory graphical resolution due to the large number of atoms in the designed molecules. In this study, the NCIs of the IDT-ED-4F and the designed derivatives were analysed using the Multiwfn 3.8 software, and the RDG isosurface plots and scatter graphs are shown in Fig. 7.

The RDG isosurfaces reveal that attractive interactions (blue) are mainly localized around electronegative atoms, while green regions indicate vdW interactions between molecular fragments, and red regions indicate steric repulsion. For IDT-ED-4F, dense blue regions indicate strong hydrogen bonding, with green regions at approximately −0.01 au indicating prominent vdW interactions, while scattered red regions represent steric clashes. In IDT1, less dense blue regions suggest weaker hydrogen bonding compared to IDT-ED-4F, with scattered green vdW interactions and fewer red steric repulsion areas. IDT2 shows dense blue regions indicating strong hydrogen bonding and green regions indicating vdW interactions, with minimal steric repulsion. The IDT3 exhibits the strong blue hydrogen bond regions in a manner similar to IDT-ED-4F, accompanied by evident vdW interactions and relatively weak red steric repulsion. IDT4 shows a few backbones with blue region (strong hydrogen bonding), some with green (vdW) and less red (steric clashes) particles. In IDT5, the dense blue colored areas (weak hydrogen bonds) and more diffuse green vdW interactions are significantly lower, and clear red steric repulsion regions can be observed. The results of RDG further confirm strong H-bonding between IDT-ED-4F and IDT4 and between IDT-ED-4F and IDT3 with slightly less steric hindrance but a similar profile. IDT1 and IDT5 reveal relatively weakened hydrogen bonding and more diffuse vdW interaction. These results demonstrate that changes to the π-bridges control the non-covalent interaction patterns of the designed molecules which correlates with the extent of planarity and stability and potential charge transfer properties of OSCs.

### Dipole moment and exciton binding energy

The dipole moment (*D*) is a fundamental parameter to determine the electron density distribution, molecular polarity and solubility, which are closely related to the OE properties of OPVs. A larger *D* enhances charge separation and results in strong separation of charge between the positive end and the negative end of the molecule, to promote efficient ICT and improve charge transport while reducing recombination losses.^61^ In this study, the dipole moments of the IDT-ED-4F and the designed derivatives were computed using the B3LYP/6-31G(d,p) level of theory. The calculated *D* values in the gaseous phase decrease in the order of IDT1 > IDT-ED-4F > IDT4 > IDT5 > IDT2 > IDT3, as presented in Table S1. The maximum *D* value of IDT1 indicates that the molecule has a higher molecular polarity, which is important for good solubility, film formation, and charge mobility, essential for high performance OSCs. Exciton binding energy (*E*_b_) is also a key parameter in OSC performance, as it represents the Coulomb binding between the electron–hole pairs and determines the dissociation as well as the free carrier generation of excitons. Smaller *E*_b_ values lower the charge separation capability, improving the energy conversion efficiency.^62^*E*_b_ values from the calculations for each of the molecules are listed in Table S5. IDT-ED-4F has *E*_b_ values of 0.20 eV in the gaseous phase and 0.33 eV in solvent. IDT2 has *E*_b_ values of 0.13 eV in the gaseous phase and 0.22 eV in the solvent, IDT3 has *E*_b_ values of 0.14 eV in the gaseous phase and 0.21 eV in the solvent, and IDT5 has *E*_b_ values of 0.16 eV in the gaseous phase and 0.27 eV in the solvent. The simultaneous analysis of *D* and *E*_b_ clearly indicates that the designed molecules, especially IDT2, IDT3 and IDT5 have desirable electronic features for efficient charge separation and transport. This clearly indicates the importance of strategic molecular engineering to adjust these parameters promoting the PV performance of OSC materials.

### Oscillator strength and excitation energy

The transition probabilities of electronic excitations are quantified by oscillator strength (*f*), while the energy required for these transitions is termed excitation energy (*E*_x_). The *f* indicates the intensity of electronic transitions and is directly related to the molecule's ability to absorb light efficiently, which is critical for enhancing the photon harvesting capability in OSCs.^63^ Higher *f* values indicate stronger absorption and greater molar absorption coefficients, contributing to improved charge transfer and photocurrent generation.^64^ In this study, the *f* and *E*_x_ for the IDT-ED-4F and the designed derivatives were calculated up to 10 excited states using TD-DFT at the B3LYP/6-31G(d,p) level of theory. However, the first excited state (S1) was found to actively contribute to the HOMO–LUMO transition with notable *f* values. For the gas phase, the *f* value of IDT-ED-4F is calculated as 2.74. Among the designed derivatives, IDT4 exhibits the highest *f* value of 2.88, followed by IDT5 (2.56) and IDT1 (2.69). The lowest *f* is observed for IDT3 with an *f* value of 1.49. The detailed values are summarized in Table S1. These results suggest that IDT4 possesses superior photon harvesting capability among the designed molecules in the gas phase. In the solvent phase (chloroform), the *f* value of IDT-ED-4F increases to 2.99. Among the designed molecules, IDT4 again demonstrates the highest *f* value (3.11), followed by IDT5 (2.86) and IDT1 (2.93). IDT3 shows the lowest *f* value in solvent with a value of 1.84, as detailed in Table S2. The overall trend indicates that all designed molecules exhibit strong *f* values, suggesting efficient light absorption and suitability for OSC applications.

Excitation energy (*E*_x_) represents the energy required for an electron to transition from the HOMO to the LUMO. Lower *E*_x_ values facilitate easier electronic excitation, promoting efficient ICT and enhancing charge transfer within the molecule. In the gas phase, the *E*_x_ of IDT-ED-4F is 1.72 eV, while the designed derivatives exhibit *E*_x_ values ranging from 1.22 eV (IDT3) to 1.84 eV (IDT4). Notably, IDT3 shows the lowest *E*_x_, indicating its superior *n*–π* transition character and efficient photoexcitation capability. In the solvent phase, the *E*_x_ value of IDT-ED-4F is 1.59 eV. Among the designed molecules, IDT3 again exhibits the lowest *E*_x_ value of 1.14 eV, followed closely by IDT2 at 1.15 eV. These low *E*_x_ values suggest that IDT3 and IDT2 require less energy for electronic transitions, supporting efficient ICT. The comprehensive *E*_x_ and *f* values for both gas and solvent phases are provided in Tables S1 and S2. The lower *E*_x_ and *f* of the designed molecules, particularly IDT3, IDT2, and IDT4, indicate their strong photon absorption and efficient charge transfer characteristics, highlighting their potential as promising active materials in high-performance OSCs.

## Photovoltaic performance parameters

### Open circuit voltage (*V*_oc_)

The open circuit voltage (*V*_oc_) is a key PV parameter representing the maximum voltage a solar cell can deliver under illumination without drawing an external current. It serves as an indicator of the energy level alignment between donor and acceptor materials. *V*_oc_ is influenced by factors such as temperature, light intensity, device architecture, and, notably, the frontier molecular orbital energies of the donor and acceptor.^65^ The energy offset between the donor HOMO and acceptor LUMO levels governs the achievable *V*_oc_. Generally, a greater offset yields a higher *V*_oc_, provided it does not compromise charge separation efficiency or increase energy losses. In this study, PTB1 is selected as the donor material, with experimentally reported HOMO and LUMO energy levels of −4.90 eV and −3.20 eV, respectively,^66,67^ while the designed IDT derivatives were assessed as acceptor materials. The theoretical *V*_oc_ values were calculated using the following equation:3
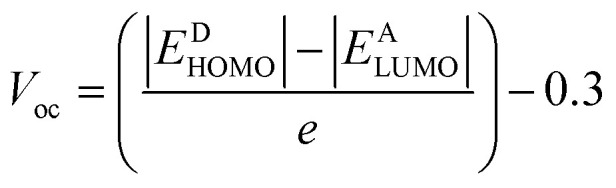


Energies are denoted by *E*, the charge on the discrete molecule is indicated by *e*, and 0.3 eV represents energy losses linked to non-radiative recombination, interfacial energy barriers, and the formation of charge transfer states.^68^

IDT-ED-4F exhibited a calculated *V*_oc_ of 1.34 V when paired with PTB1. Among the designed derivatives, *V*_oc_ values followed the order: IDT1 > IDT-ED-4F > IDT4 > IDT5 > IDT2 > IDT3, with IDT1 showing the highest *V*_oc_ at 1.40 V. This enhanced *V*_oc_ for IDT1 arises due to its lower LUMO energy level compared to other derivatives, which results in a greater energy offset between the HOMO of PTB1 (donor) and the LUMO of IDT1 (acceptor). This larger offset facilitates higher photovoltage generation without adversely affecting the charge separation efficiency. In contrast, IDT2 and IDT3 exhibited comparatively lower *V*_oc_ values (0.56 eV and 0.50 eV, respectively), which can be attributed to their relatively higher LUMO energy levels. These shallower LUMO levels reduce the energy difference with the PTB1 HOMO, thereby limiting the achievable *V*_oc_ despite potentially efficient ICT characteristics. The *V*_oc_ values observed for IDT4 and IDT5 suggest that while these molecules possess efficient charge transfer pathways and strong acceptor characteristics, their electronic configurations may not maximize photovoltage output due to less optimal LUMO positioning relative to PTB1. Table S6 visually presents these *V*_oc_ values, highlighting the superior performance of IDT1 compared to the IDT-ED-4F. These results confirm that the designed IDT derivatives maintain favourable energy level alignment with PTB1, with IDT1 standing out as the most promising acceptor candidate for integration into high efficiency OSCs. Its deep LUMO energy level ensures both efficient charge transfer and maximized photovoltage output, essential for achieving superior PV device performance.

### Fill factor (FF)

Fill factor (FF) directly impacts the overall power conversion efficiency (PCE) of OSCs. It reflects the extent to which a device approaches its maximum power output and is influenced by the quality of the donor–acceptor interface, internal resistance, charge carrier mobility, and recombination dynamics. A higher FF indicates more efficient extraction of photogenerated charge carriers and minimal losses in charge transport. It is also strongly dependent on the *V*_oc_, as it correlates with the built-in potential of the active layer and the capacity to sustain photogenerated charge separation under operational conditions.^69,70^ The FF was estimated using a semi-empirical relationship derived from the normalized open-circuit voltage:4
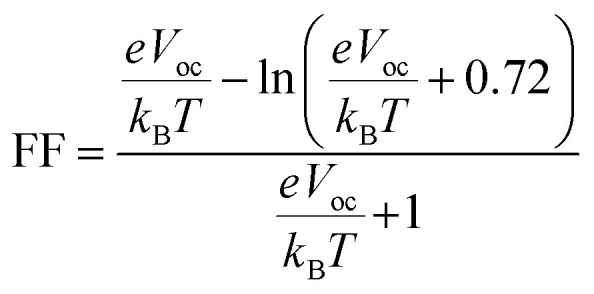
Here, *V*_oc,_*K*_B_, *T* and *e* are the open circuit voltage, the Boltzmann constant, the temperature at 300 K and the elementary charge of a molecule. The calculated FF values for *R* and designed molecules are listed in Table S6. Among the studied molecules, IDT1 exhibited the highest FF value of 90.93%, followed closely by IDT-ED-4F (90.61%) and IDT4 (90.50%). The FF values for all molecules in increasing order are: IDT3 (80.34%) < IDT2 (81.39%) < IDT5 (87.94%) < IDT4 (90.50%) < IDT-ED-4F (90.61%) < IDT1 (90.93%). The superior FF of IDT1 can be attributed to its optimal energy level alignment with the donor PTB1, facilitating reduced recombination losses and efficient extraction of photogenerated charge carriers. This high FF reflects enhanced built-in electric field strength and effective charge collection, both critical for achieving superior PV performance. In contrast, IDT2 and IDT3 exhibited the lowest FF values, likely due to their lower *V*_oc_ and less favourable energy offsets, which can lead to increased charge recombination and reduced overall device efficiency. The results confirm that IDT1, IDT-ED-4F, and IDT4 maintain excellent charge transport and collection characteristics, making them particularly promising candidates for integration into high efficiency OSC devices. Beyond their photovoltaic properties, solubility and film forming ability are also important for practical device fabrication. The incorporation of heterocyclic π-linkers and electron-withdrawing groups in the designed molecules introduces polarity into the molecular framework, which is generally favourable for solubility in common organic solvents. In particular, the relatively large *D* values predicted for several derivatives (*e.g.*, IDT1) suggest improved interactions with polarizable solvents and potentially better processability compared to highly rigid fused-ring acceptors. These structural features indicate that the designed molecules are not only promising from an optoelectronic perspective but may also offer advantages in solution based processing routes frequently employed in OSC fabrication.

### Charge transfer efficiency at the PTB1–IDT interface

As established in the preceding sections, the designed IDT derivatives demonstrated improved OE properties, including enhanced absorption characteristics, reduced reorganization energies, and favourable frontier molecular orbital alignments, suggesting their potential as efficient acceptor materials in OSCs. However, beyond these intrinsic properties, evaluating the charge transfer (CT) dynamics at the donor–acceptor interface is essential to predict their practical PV performance. Efficient CT is a pivotal factor in determining the overall device efficiency of OSCs and is influenced by parameters such as molecular planarity, π–π stacking, and electronic coupling between donor and acceptor moieties. Among the designed derivatives, IDT1 emerged as the optimal candidate for CT analysis based on its superior PV parameters, including the highest PCE, favourable *V*_oc_, and strong oscillator strength. A donor–acceptor complex of PTB1 : IDT1 was constructed for further analysis, with the geometry optimized at the B3LYP/6-31G(d,p) level of theory to accurately predict interfacial electronic distributions. The acceptor moiety of IDT1 was strategically oriented towards PTB1 to maximize orbital overlap and facilitate efficient electronic communication.

**Fig. 8:**
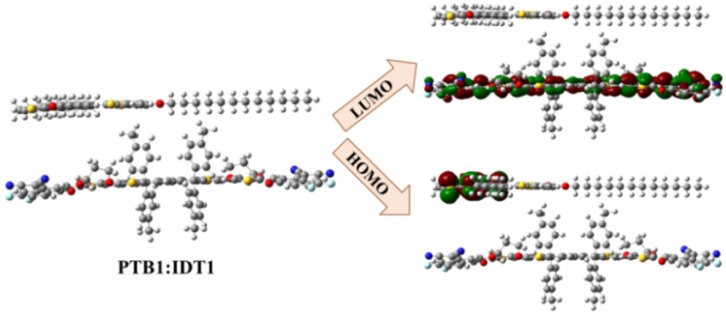
Charge transfer exploration in PTB1 donor and IDT1.

The charge density distribution, illustrated in Fig. 8, reveals a clear localization of the HOMO on the PTB1 donor polymer and the LUMO on the IDT1 acceptor molecule. This distinct separation indicates a favourable CT pathway upon excitation, enabling efficient exciton dissociation and minimizing recombination losses. The pronounced electronic delocalization toward the IDT1 acceptor underscores its role in enhancing the external quantum efficiency and overall photocurrent generation of the device. These findings further validate the structural and electronic design strategies adopted in this study, confirming IDT1 as a promising acceptor candidate for high efficiency OSCs due to its ability to effectively harness absorbed photons, facilitate rapid charge separation, and promote efficient carrier collection in practical device configurations.

## Conclusions

In summary, this study presents a comprehensive investigation into the molecular design of A-π-D-π-A structured molecules to enhance the PV performance of OSCs. Five novel IDT-based derivatives (IDT1–IDT5) were systematically designed by incorporating diverse π-bridges and evaluated using DFT and TD-DFT methods to explore their OE properties and device potential. Our findings demonstrate that IDT1 exhibits outstanding PV characteristics, including an optimal *V*_oc_ of 1.40 V and the highest FF of 90.93%, indicating efficient charge collection and minimal recombination losses. IDT3 displayed the smallest *E*_g_ of 1.35 eV, suggesting superior light absorption and enhanced charge carrier generation. Furthermore, IDT3 showed the lowest *λ* (*λ*_e_ = 0.00578 eV and *λ*_h_ = 0.00503 eV), confirming its excellent charge mobility and structural reorganization efficiency during charge transfer processes. The dipole moment analysis revealed that IDT1 exhibited the highest dipole moments in both gas and solvent phases, supporting its strong charge separation and solubility properties, which are essential for effective film formation and interfacial interactions in OSC devices. *E*_b_ calculations indicated that IDT3 and IDT2 possess the lowest *E*_b_ values (0.14 eV and 0.13 eV, respectively), signifying a higher tendency for exciton dissociation, which is favourable for enhancing photocurrent generation and the overall device efficiency. TDM and DOS analyses confirmed the efficient ICT and substantial LUMO localization on the acceptor units, further highlighting their potential as active layers for OSCs. NCI and RDG analyses demonstrated stabilizing interactions within their molecular frameworks, enhancing planarity and π–π stacking, which are critical for charge transport. Overall, all newly designed molecules (IDT1–IDT5) exhibited reduced *E*_g,_ redshifted absorption, and enhanced charge transport properties compared to the reference IDT-ED-4F molecule. These findings provide valuable insights for rational molecular engineering and pave the way for developing next generation OSCs with superior efficiency, stability, and device performance.

## Author contributions

S. M. K. A. Naqvi and R. A. Khera conceived and designed the study; H. Naeem, T. A. Alrebdi, K. H. Salem, and M. Imran carried out the computational modeling and figure preparation; A. Almohammedi and M. S. Soliman contributed to data analysis and interpretation; H. Naeem and M. Faizan participated in writing and editing of the manuscript.

## Conflicts of interest

The authors declare that they have no known competing financial interests or personal relationships that could have appeared to influence the work reported in this paper.

## Supplementary Material

NA-OLF-D5NA00680E-s001

NA-OLF-D5NA00680E-s002

NA-OLF-D5NA00680E-s003

NA-OLF-D5NA00680E-s004

NA-OLF-D5NA00680E-s005

## Data Availability

All data provided and/or analysed during this study were included as figures and tables in this article and its supplementary information (SI). Supplementary information is available. See DOI: https://doi.org/10.1039/d5na00680e.
