# Supplementary figures and images for: Heterocyclic π-linkers for reduced energy dissipation in symmetrical IDT-core-based non-fullerene acceptors: a route to efficient organic solar cells
Source: Nanoscale Adv. 2025 Oct 16;7(23):7681–93. doi: 10.1039/d5na00680e (PMC12529793; doi:10.1039/d5na00680e)

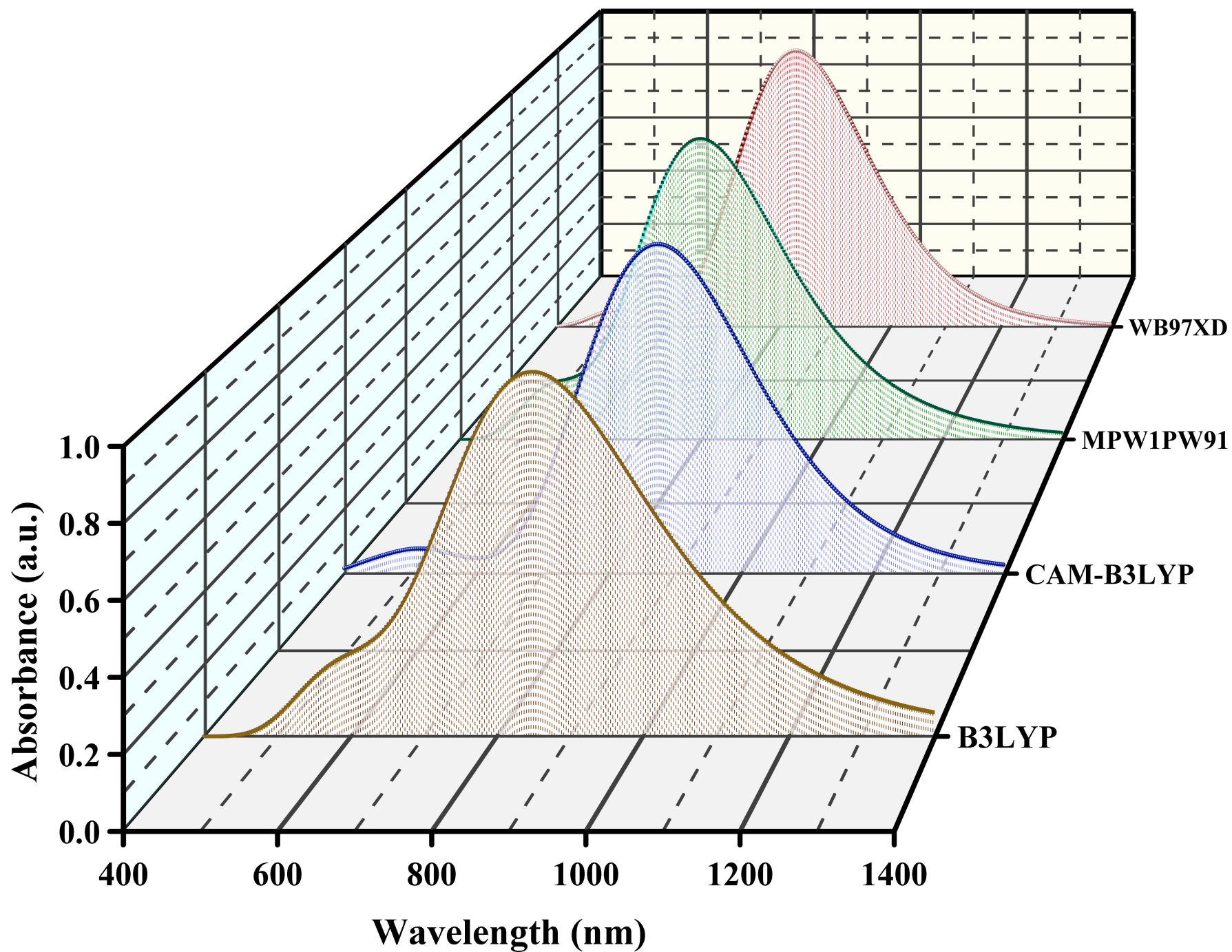

Supplement: NA-007-D5NA00680E-s002 [file NA-007-D5NA00680E-s002.pdf]

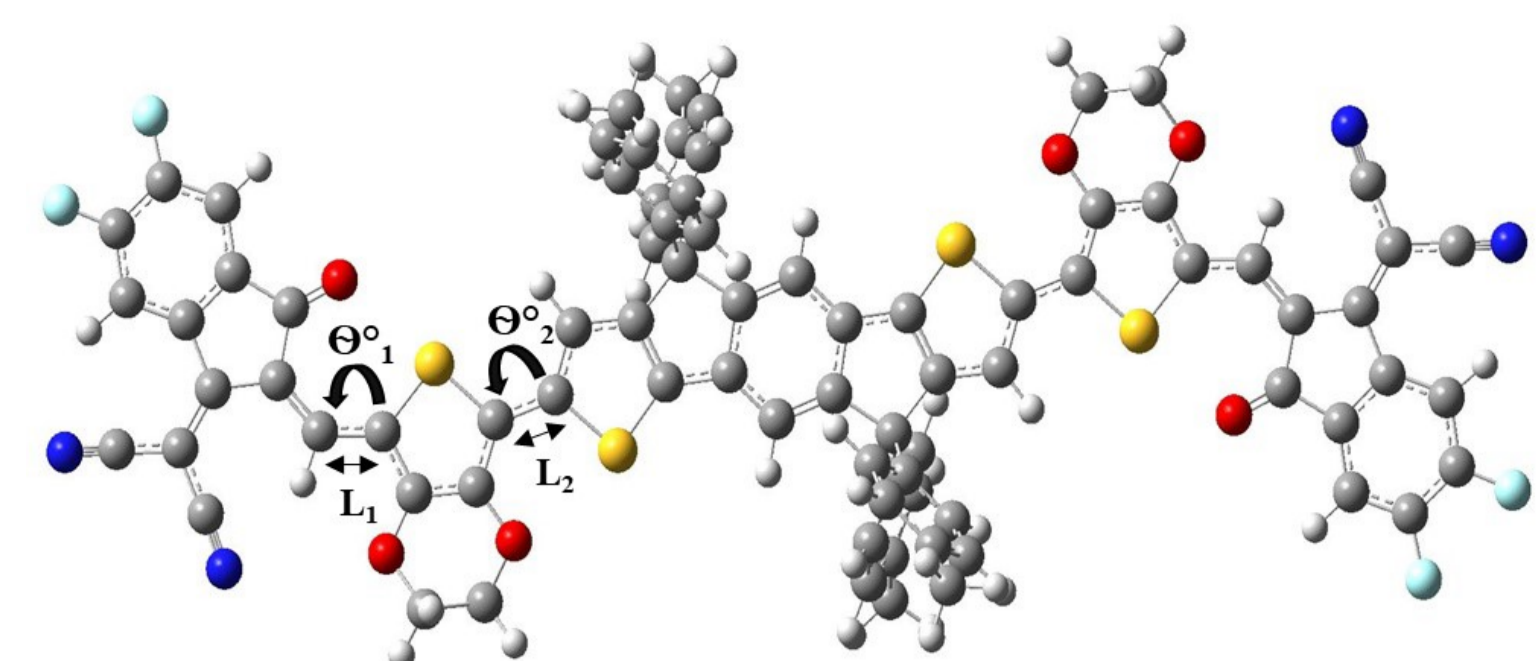

**IDT-ED-4F**

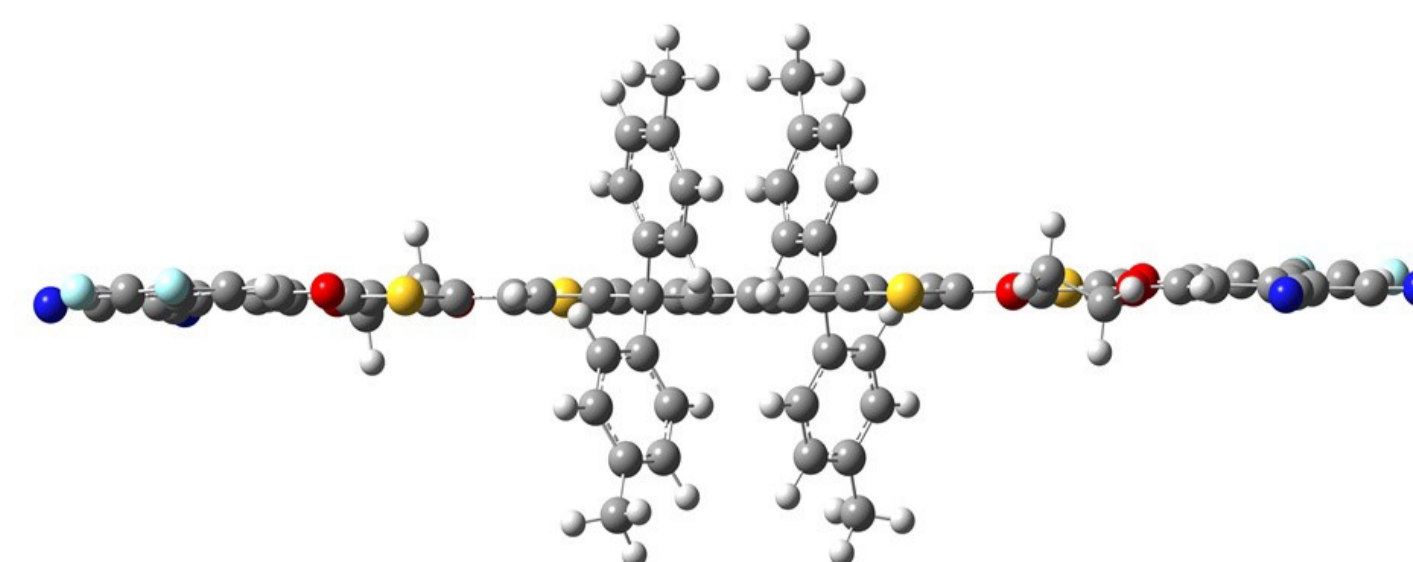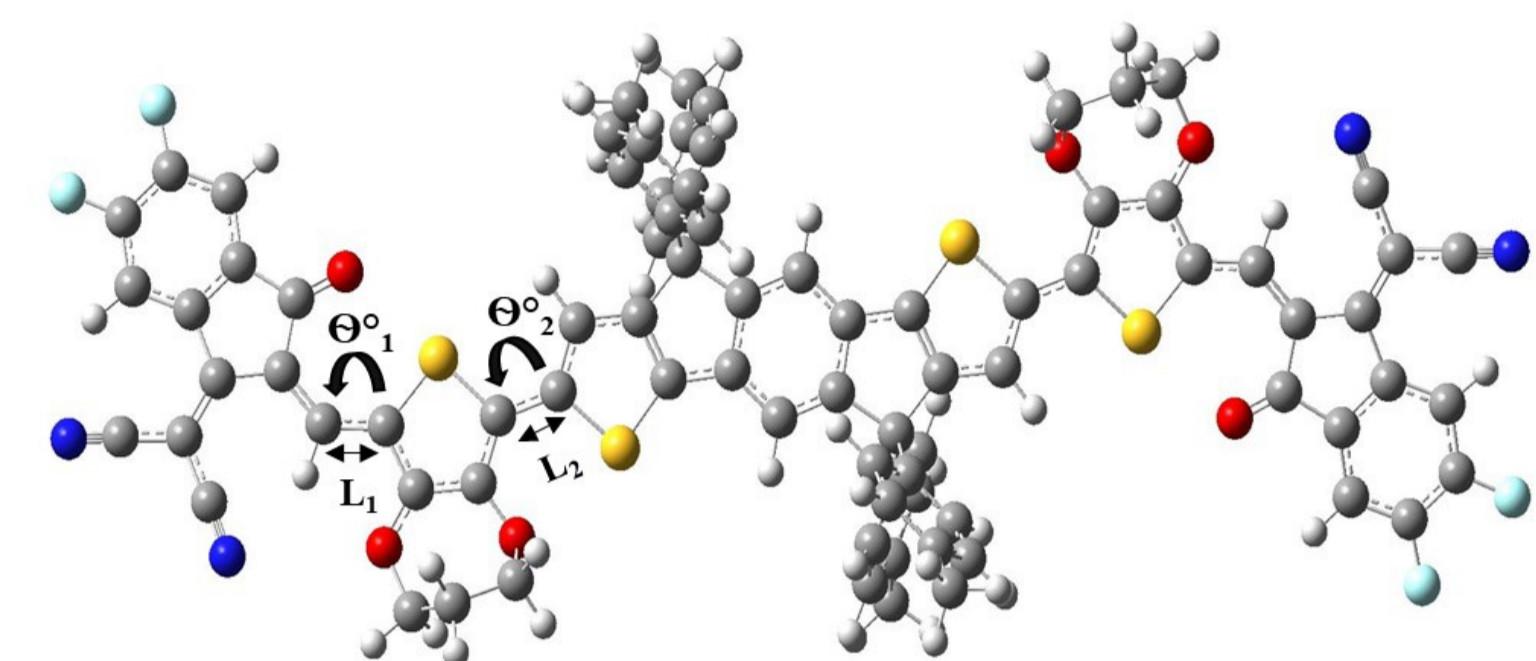

**IDT1**

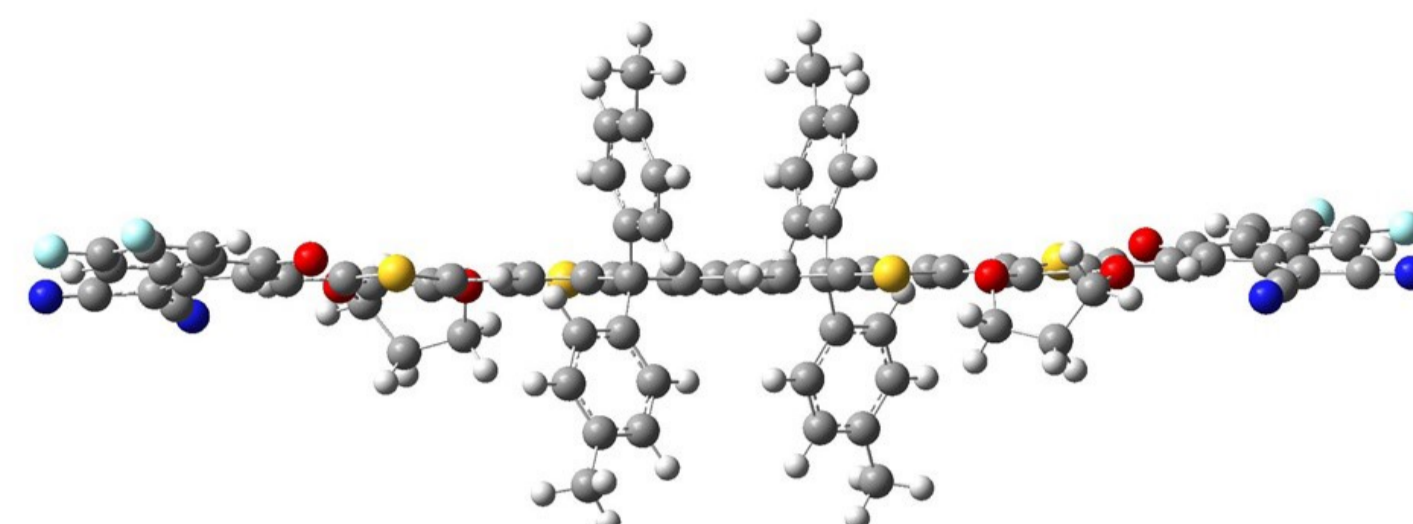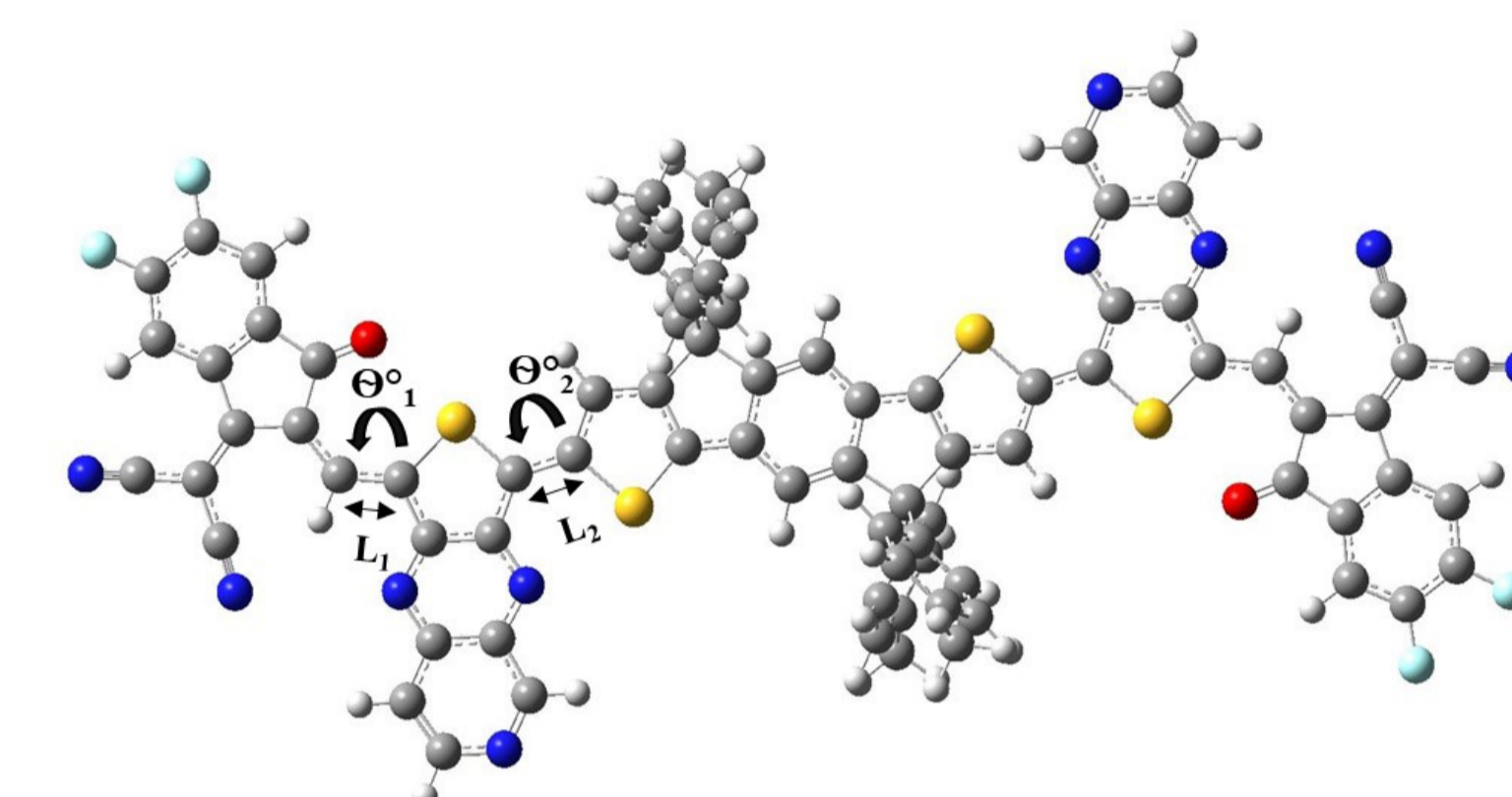

**IDT2**

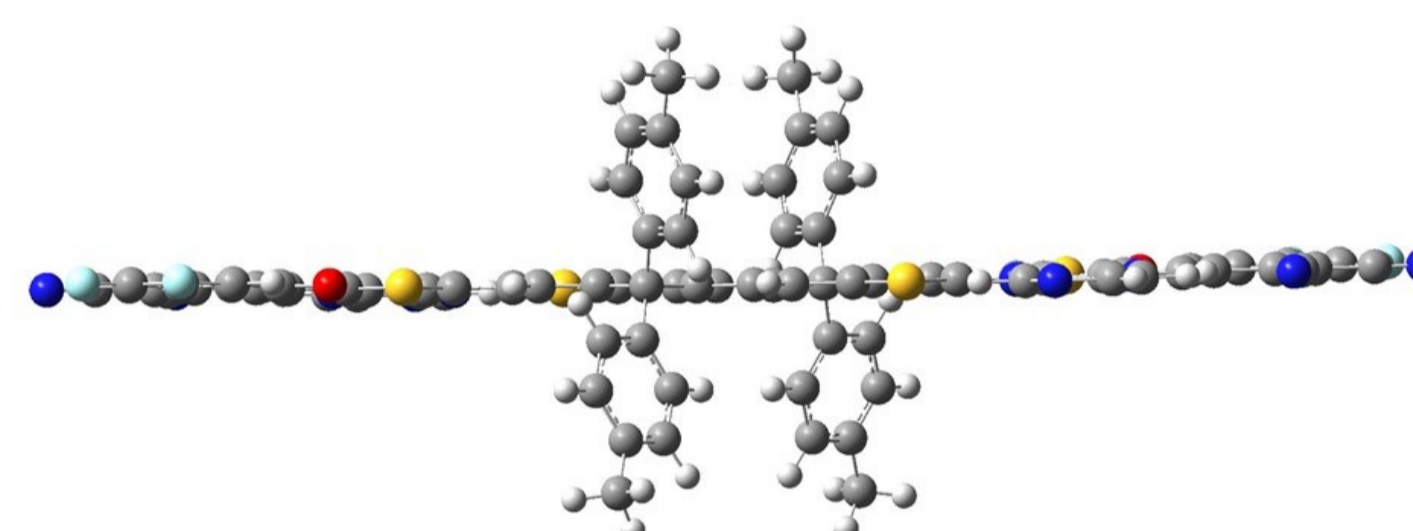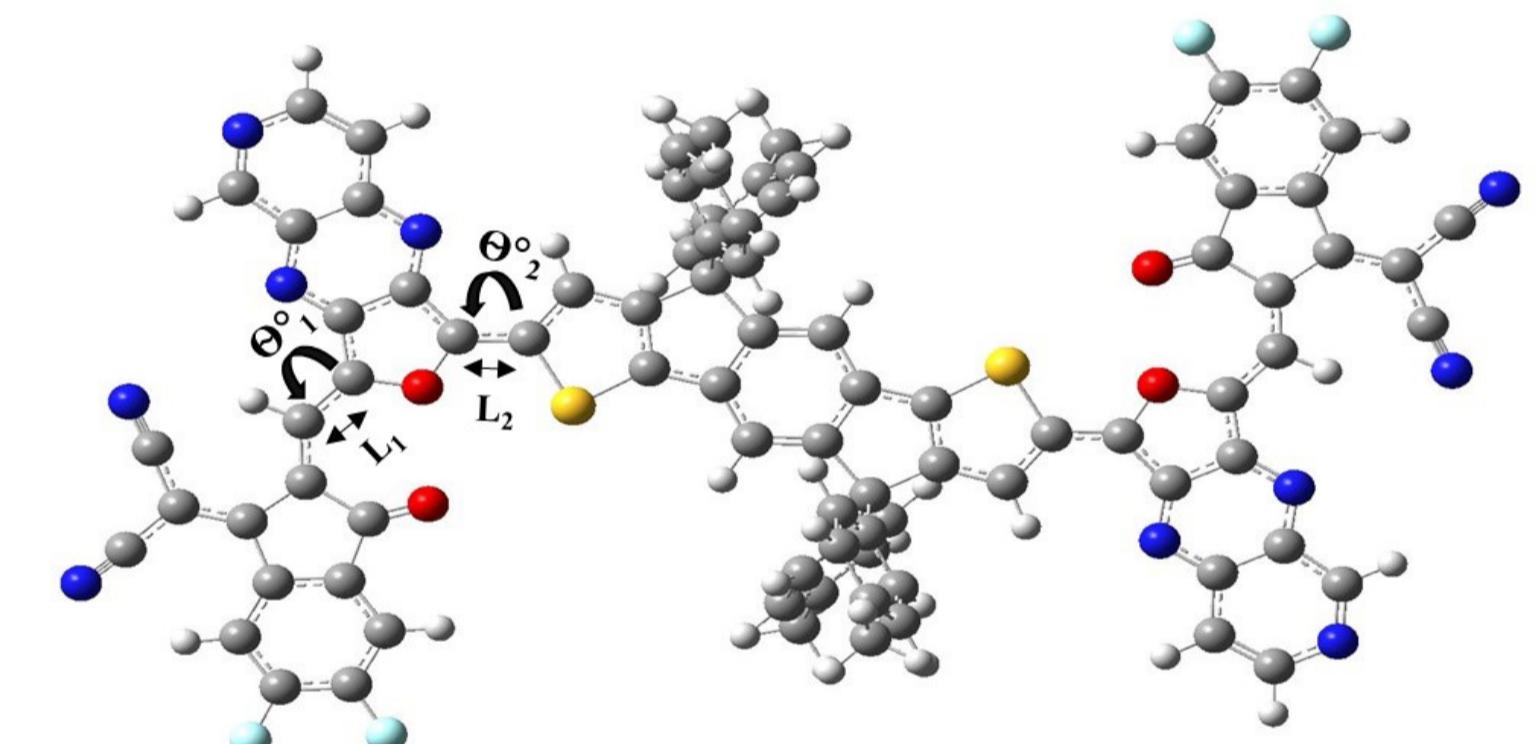

**IDT3**

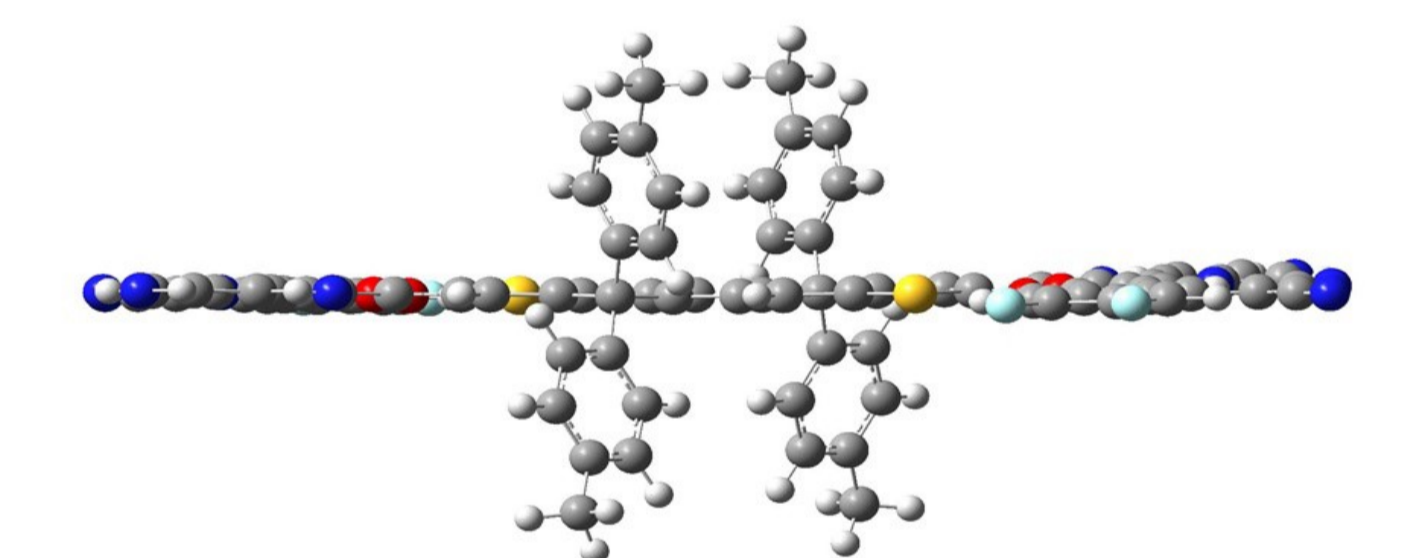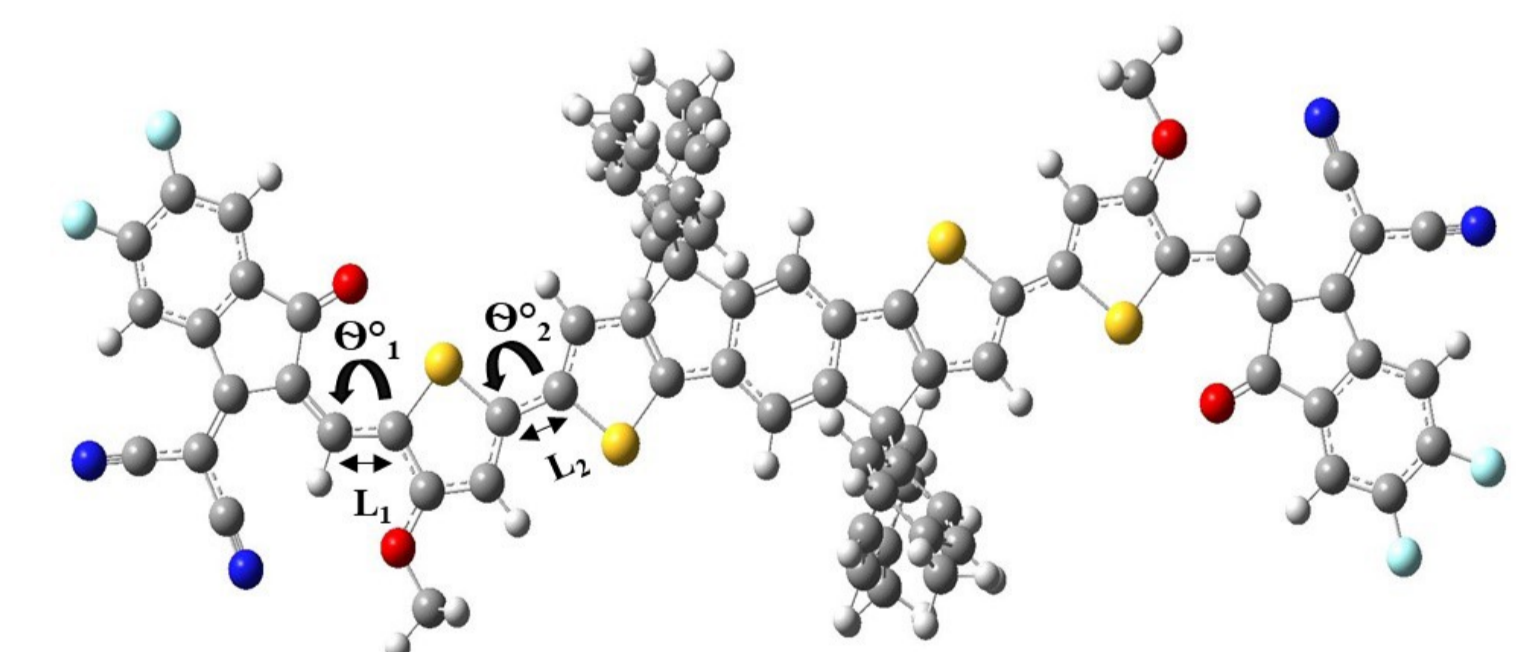

**IDT4**

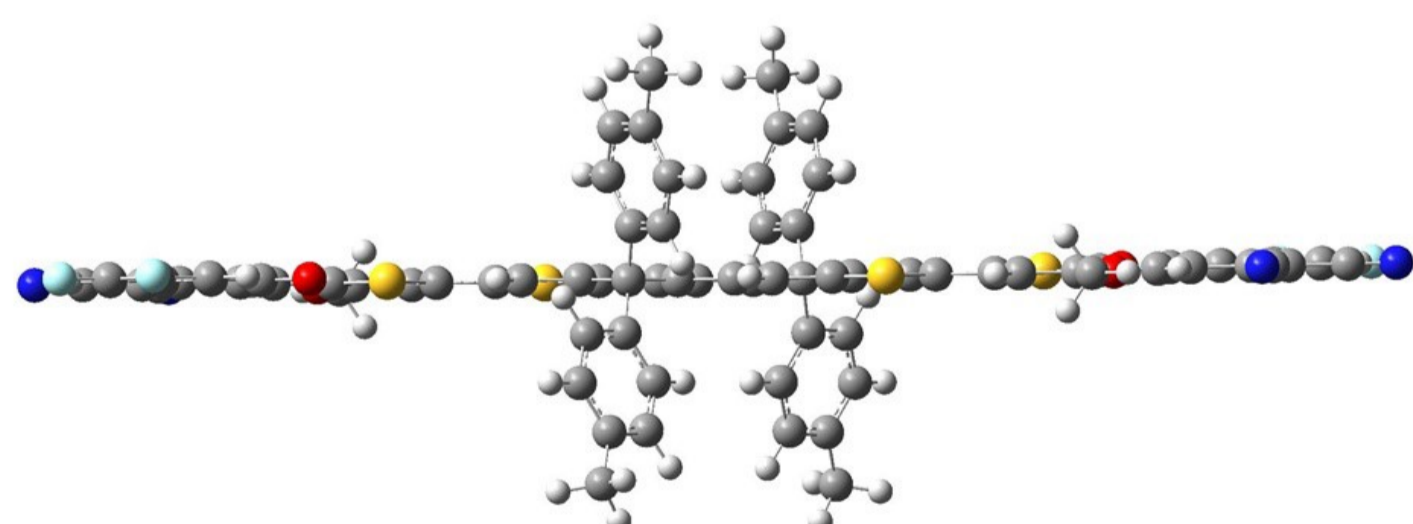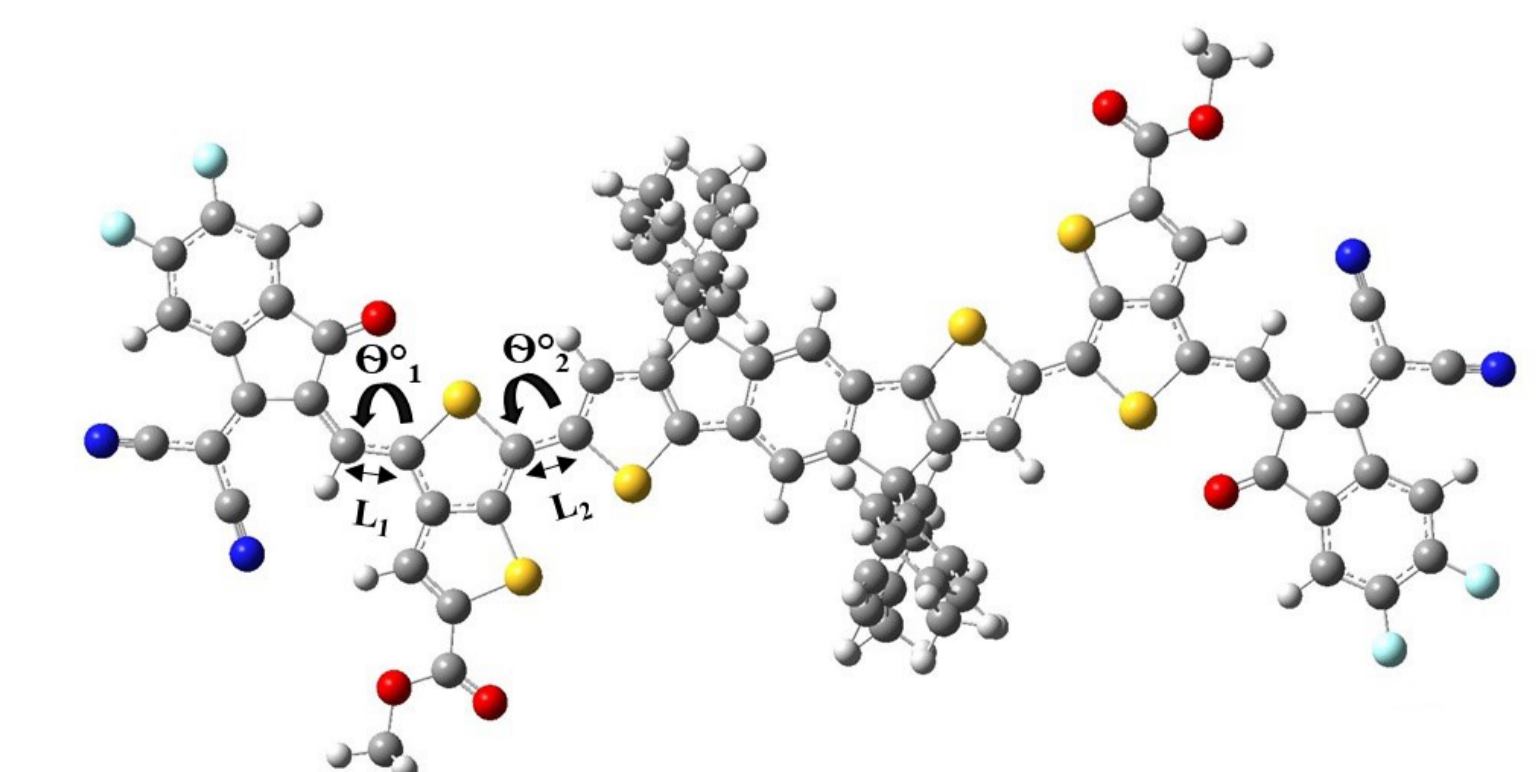

**IDT5**

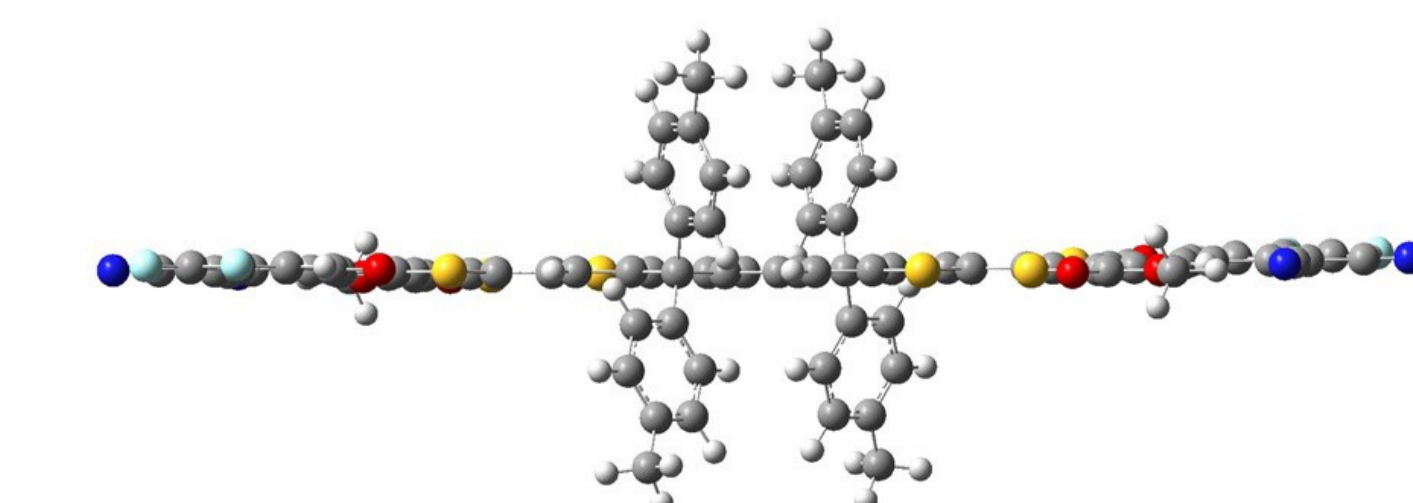

Supplement: NA-007-D5NA00680E-s003 [file NA-007-D5NA00680E-s003.pdf]

**IDT-ED-4F**

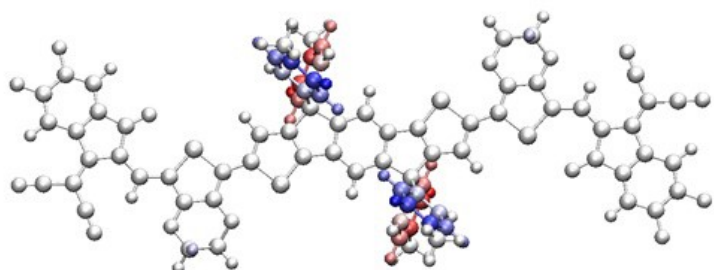

**MPP=1.545 Å**

**SDP=9.097 Å**

**IDT1**

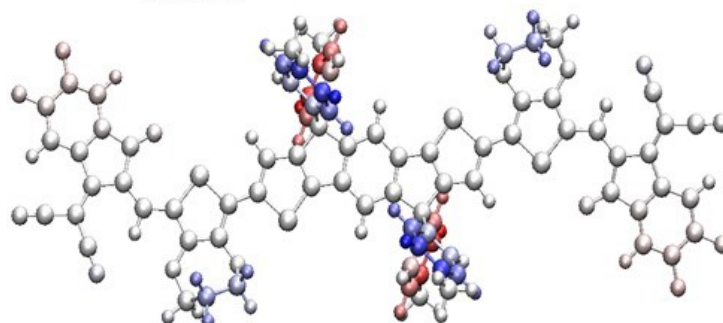

**MPP=1.624 Å**

**SDP=9.103 Å**

**IDT2**

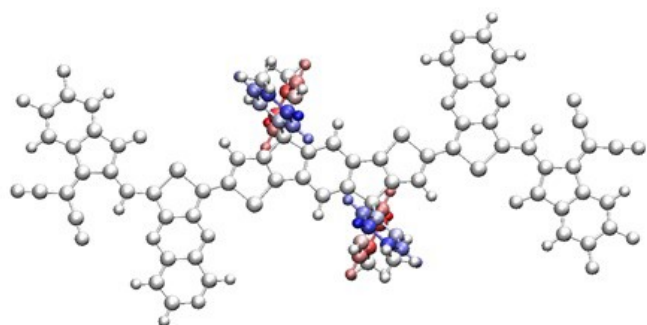

**MPP=1.489 Å**

**SDP=9.113 Å**

**IDT3**

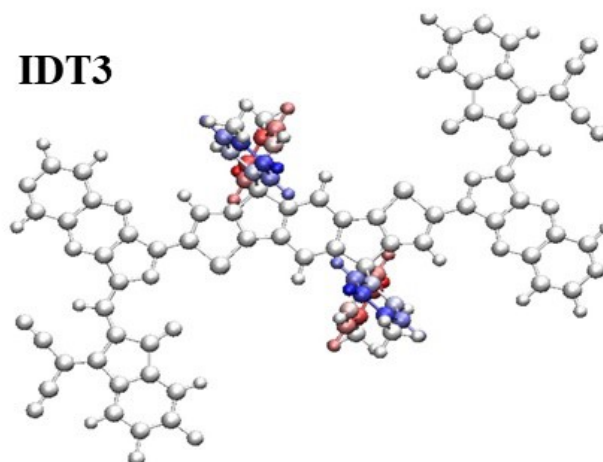

**MPP=1.488 Å**

**SDP=9.113 Å**

**IDT4**

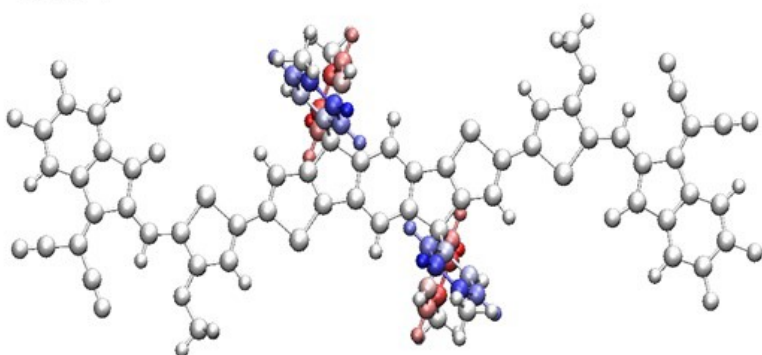

**MPP=1.625 Å**

**SDP=9.104 Å**

**IDT5**

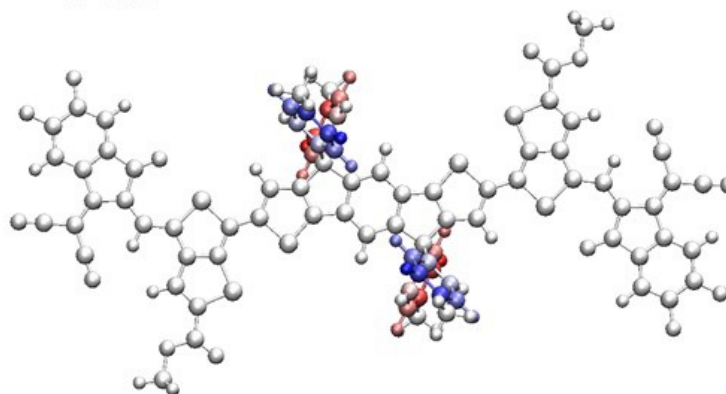

**MPP=1.535 Å**

**SDP=9.106 Å**

Supplement: NA-007-D5NA00680E-s004 [file NA-007-D5NA00680E-s004.pdf]
