# Supplementary material for: Heterocyclic π-linkers for reduced energy dissipation in symmetrical IDT-core-based non-fullerene acceptors: a route to efficient organic solar cells
Source: Nanoscale Adv. 2025 Oct 16;7(23):7681–93. doi: 10.1039/d5na00680e (PMC12529793; doi:10.1039/d5na00680e)
Supplement: NA-007-D5NA00680E-s005 [file NA-007-D5NA00680E-s005.pdf]

Supplementary Information (SI) for Nanoscale Advances.
This journal is © The Royal Society of Chemistry 2025

Supporting Information

Heterocyclic π-Linkers for Reduced Energy Dissipation in Symmetrical IDT-Core Based Non-Fullerene Acceptors: A Route to Efficient Organic Solar Cells

Hina Naeem,[a] Tahani A. Alrebdi,[b] Karrar Hazim Salem,[c] Muhammad Imran,[d] Abdullah Almohammedi,[e] Mohamed S. Soliman,[f] Hira Naeem,[g] Muhammad Faizan,[h] Syed Muhammad Kazim Abbas Naqvi *[i,j] and Rasheed Ahmad Khera *[a]

[a]Department of Chemistry, University of Agriculture, Faisalabad 38000, Pakistan
[b]Department of Physics, College of Science, Princess Nourah bint Abdulrahman University, P.O. Box 84428, Riyadh 11671, Saudi Arabia
[c]College of Medical and Health Technologies, Al-Zahraa University for Women, Karbala, Iraq
[d]Department of Chemistry, Faculty of Science, Research Center for Advanced Materials Science (RCAMS), King Khalid University, P.O. Box 960, Abha, 61421, Saudi Arabia
[e]Department of Physics, Faculty of Science, Islamic University of Madinah, Madinah, Saudi Arabia
[f]Department of Electrical Engineering, College of Engineering, Taif University, Taif, 21944, Saudi Arabia
[g]Department of Chemistry, GC-Women University, Faisalabad
[h]School of Materials Science and Engineering Jilin University, Changchun, China
[i]Faculty of Materials Science, Shenzhen MSU-BIT University, Shenzhen, 518115, China
[j]Platform for Applied Nanophotonics, Institute of Advanced Interdisciplinary Technology, Shenzhen MSU-BIT University, Shenzhen, 518115, China

List of Figures

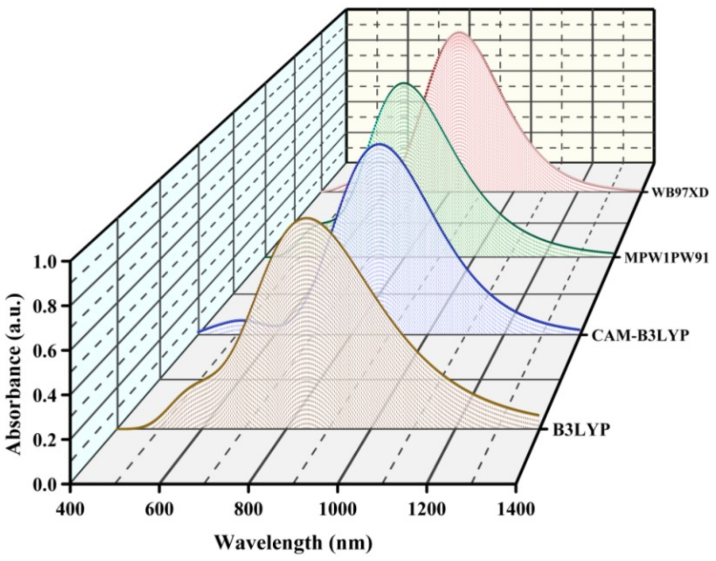

Fig. S1 Comparison of four distinct functionals (B3LYP, CAMB3LYP, WB97XD, and MPW1PW91)

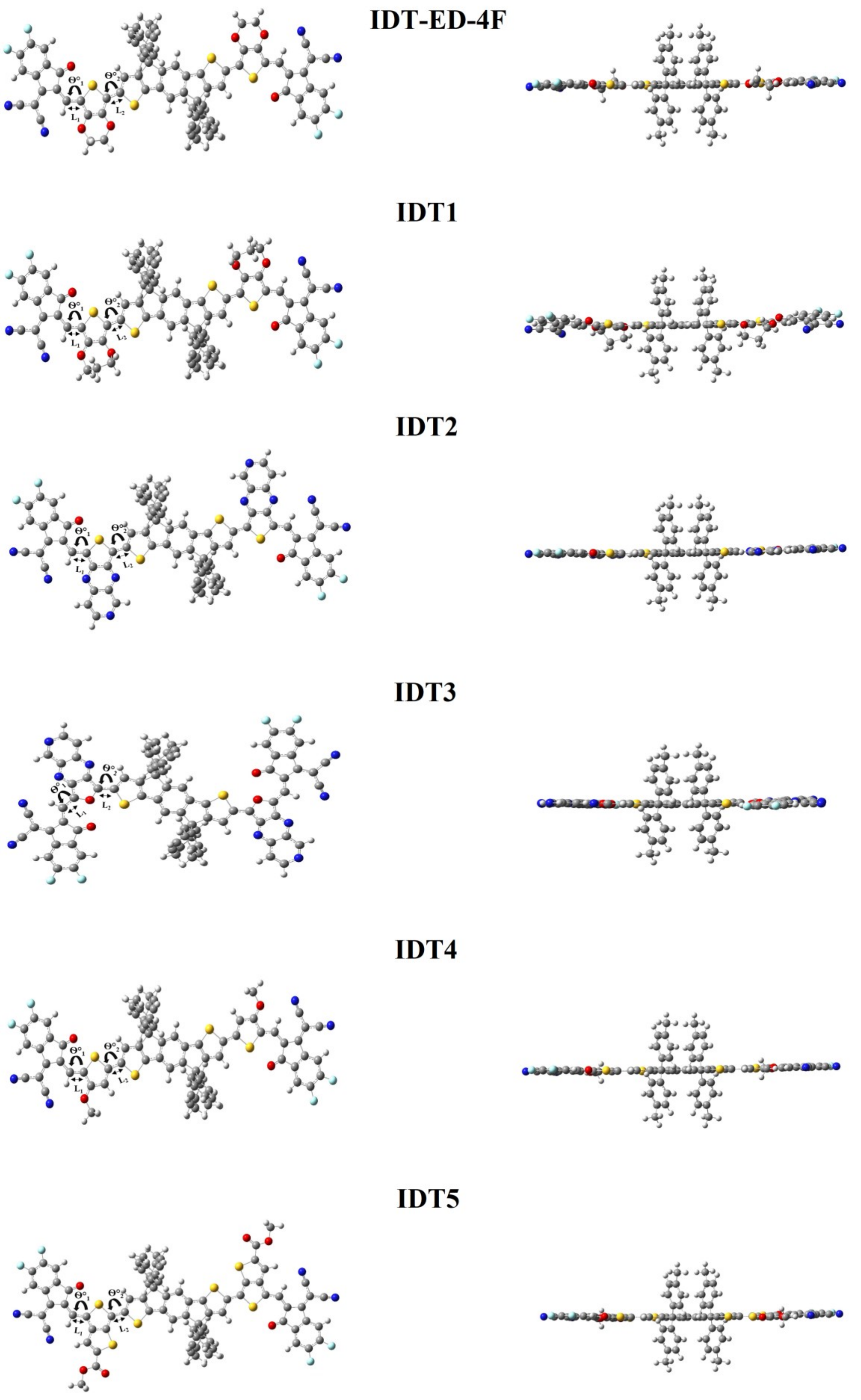

Fig. S2 Optimized geometries of IDT-ED-4F and IDT1-IDT5 illustrating interatomic distances and torsional angles

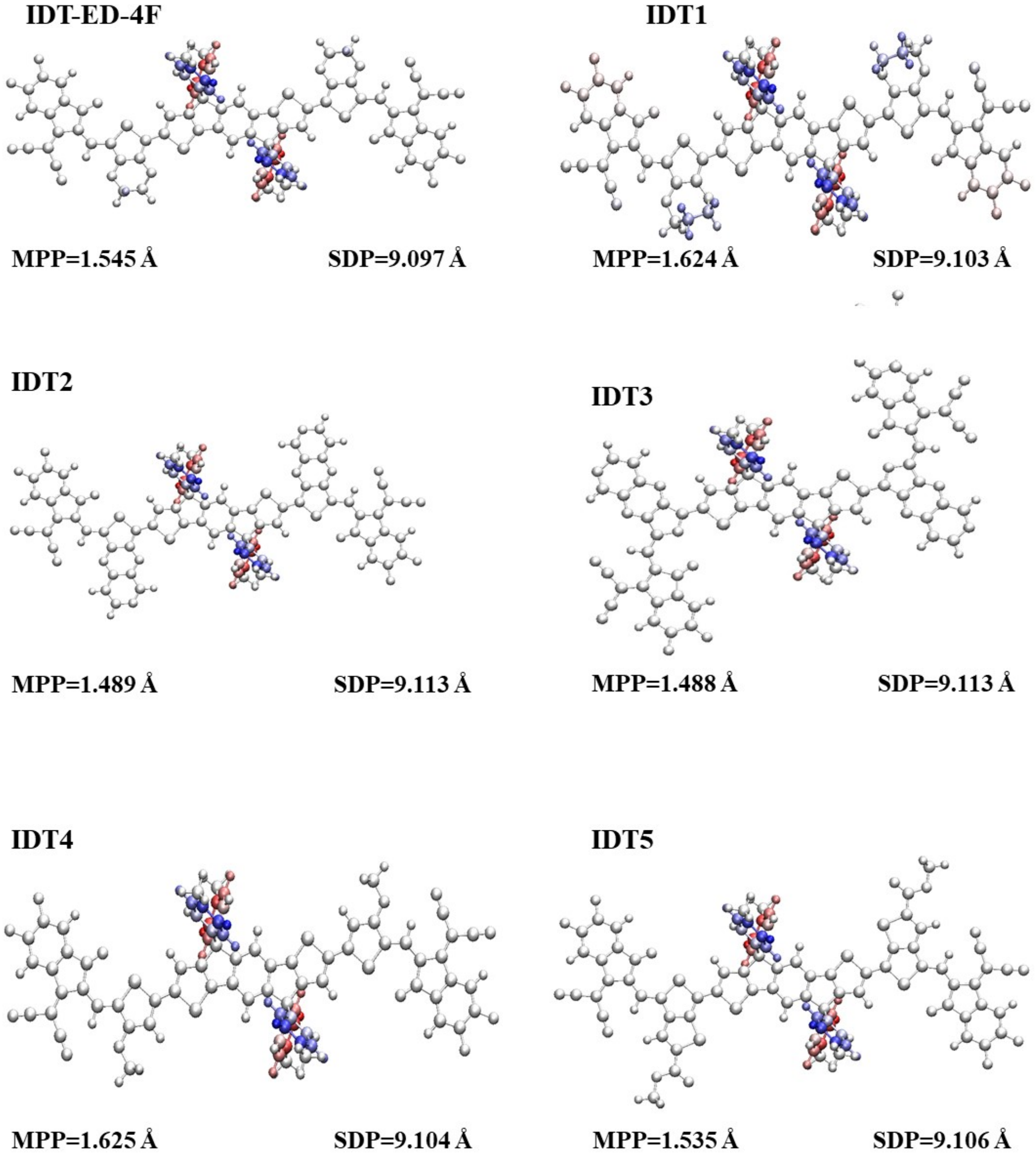

Fig. S3 Visualization of molecular planarity in IDT-ED-4F and IDT1-IDT5 based on MPP and SDP values

List of Tables

Table S1 Calculated absorption profile (λ_{max}), light harvesting efficiency (LHE), Excitation energy (E_X), Oscillating strength (f), and Dipole moments (D) for IDT-ED-4F and IDT1-IDT5 in gas phase

Molecule	Experimental λ_{max} (nm)	Calculated λ_{max} (nm)	Excitation energy (E_x) (eV)	Oscillator strength (f)	Interaction Coefficient	Dipole moment (D)
IDT-ED-4F	751	719	1.72	2.74	0.7040	0.4250
IDT1	-	694	1.79	2.69	0.7023	2.6700
IDT2	-	998	1.24	1.96	0.7042	0.0715
IDT3	-	1020	1.22	1.49	0.7037	0.0094
IDT4	-	674	1.84	2.88	0.7015	0.2344
IDT5	-	792	1.56	2.56	0.7067	0.0738

Table S2 Calculated absorption profile (λ_{max}), light harvesting efficiency (LHE), Excitation energy (E_X), Oscillating strength (f), and Dipole moments (D) for IDT-ED-4F and IDT1-IDT5 in solvent

Molecule	Experimental λ_{max} (nm)	Calculated λ_{max} (nm)	Excitation energy (E_x) (eV)	Oscillator strength (f)	Interaction Coefficient	Dipole moment (D)
IDT-ED-4F	751	777	1.59	2.99	0.7001	0.5190
IDT1	-	746	1.66	2.93	0.6983	3.1720
IDT2	-	1080	1.15	2.29	0.7018	0.1020
IDT3	-	1087	1.14	1.84	0.7020	0.0579
IDT4	-	724	1.71	3.11	0.6970	0.2930
IDT5	-	859	1.44	2.86	0.7030	0.0830

Table S3 Theoretically calculated HOMO, LUMO and bandgap (E_g) values of IDT-ED-4F and designed molecules using the B3LYP/6-31G(d,p) level of theory

Molecule	E_{HOMO} (eV)	E_{LUMO} (eV)	E_g (eV)
IDT-ED-4F	- 5.19	- 3.26	1.92
IDT1	- 5.22	- 3.20	2.02
IDT2	-5.43	- 4.06	1.37
IDT3	- 5.45	- 4.10	1.35
IDT4	- 5.36	- 3.28	2.07
IDT5	- 5.35	- 3.63	1.72

Table S4 Reorganization energy of electron (λ_e) and hole (λ_h) of IDT series

Molecule	λ_e (electron)	λ_h (hole)
IDT-ED-4F	0.0053796	0.0069566
IDT1	0.0057567	0.0072283
IDT2	0.0060344	0.0051637
IDT3	0.0057800	0.0050298
IDT4	0.0064633	0.0059063
IDT5	0.0246798	0.0132124

Table S5 Exciton binding energy (E_b) of IDT series in gas and solvent phase

Molecule	E_{H-L} (eV)	E_b (eV) Gaseous	E_b (eV) Solvent
IDT-ED-4F	1.92	0.20	0.33
IDT1	2.02	0.23	0.36
IDT2	1.37	0.13	0.22
IDT3	1.35	0.14	0.21
IDT4	2.07	0.23	0.36
IDT5	1.72	0.16	0.27

Table S6 Calculated fill factor (FF) and open circuit voltage (V_{oc}) for the IDT-ED-4F and the designed molecules

**Molecule**	**V_{oc} (eV)**	**FF**
IDT-ED-4F	1.34	90.61%
IDT1	1.40	90.93%
IDT2	0.56	81.93%
IDT3	0.50	80.39%
IDT4	1.32	90.50%
IDT5	0.97	87.94%
